# Recent Progress on Organic Electrodes Materials for Rechargeable Batteries and Supercapacitors

**DOI:** 10.3390/ma12111770

**Published:** 2019-05-31

**Authors:** Alain Mauger, Christian Julien, Andrea Paolella, Michel Armand, Karim Zaghib

**Affiliations:** 1 Sorbonne Université, Institut de Minéralogie, de Physique des Matériaux et de Cosmochimie (IMPMC), UMR-CNRS 7590, 4 place Jussieu, 75005 Paris, France; alain.mauger@upmc.fr (A.M.); christian.julien@courriel.upmc.fr (C.J.); 2 Centre of Excellence in Transportation Electrification and Energy Storage (CETEES), Hydro-Québec, 1806, Lionel-Boulet blvd., Varennes, QC J3X 1S1, Canada; paolella.andrea2@ireq.ca; 3 CIC Energigune, Parque Tecnol Alava, 01510 Minano, Spain; michel.armand@gmail.com

**Keywords:** organic cathode, organic anode, lithium batteries, naphtoquinones derivatives, supercapacitor

## Abstract

Rechargeable batteries are essential elements for many applications, ranging from portable use up to electric vehicles. Among them, lithium-ion batteries have taken an increasing importance in the day life. However, they suffer of several limitations: safety concerns and risks of thermal runaway, cost, and high carbon footprint, starting with the extraction of the transition metals in ores with low metal content. These limitations were the motivation for an intensive research to replace the inorganic electrodes by organic electrodes. Subsequently, the disadvantages that are mentioned above are overcome, but are replaced by new ones, including the solubility of the organic molecules in the electrolytes and lower operational voltage. However, recent progress has been made. The lower voltage, even though it is partly compensated by a larger capacity density, may preclude the use of organic electrodes for electric vehicles, but the very long cycling lives and the fast kinetics reached recently suggest their use in grid storage and regulation, and possibly in hybrid electric vehicles (HEVs). The purpose of this work is to review the different results and strategies that are currently being used to obtain organic electrodes that make them competitive with lithium-ion batteries for such applications.

## 1. Introduction

Lithium-ion batteries (LIBs) are commercially available and used worldwide. Particularly, they are strongly expanding with the development of electric vehicles (EVs) and plug-in electric vehicles (PEVs). Their anode is graphitic carbon (even though a few commercial Li-ion cells use lithium titanate Li_4_Ti_5_O_12_ anodes), but the active element of the cathode is an inorganic material that includes at least one transition metal ion (Fe, Mn, Ni, Co) in lamellar compounds, spinels, or olivine structures in its chemical formula [1]. Constant progress in reducing the cost and improving the performance of LIBs is evident, but this technology still suffers from limitations that motivate research regarding different chemistries. The main difficulty is safety, except in the case of olivine-based cathodes [2]. Another issue is that they are the source of greenhouse gas emission. The fabrication of batteries with inorganic cathodes is costly due to carbon-based gas emission, starting from the extraction of the transition metals in ores with low metal content. Overall, the fabrication of an electric car today requires the emission of twice as much carbon-based gas as a classical vehicle [3].

Organic-based electrode (OBE) materials have mainly appeared in the early 2000’s and they are considered more and more actively as possible contenders for the replacement of inorganic materials at both the positive or negative electrodes. They have several advantages, such as higher specific capacities and reduced problems with swelling upon cycling (especially when they are amorphous) and the possibility of producing flexible materials [4]. In addition, most of them are environmentally benign and less inexpensive (depending, however, of the synthesis). Nevertheless, some of them are highly toxic (for example, viologens). The replacement of inorganic electrodes with abundant organic materials is desirable, not only for reducing the price of LIBs, but also for reducing the CO_2_ footprint [5,6,7].

Nature and biology give many examples of organic molecules that are suitable. For example, quinones play a key role in the photosynthesis process [8]. The cells metabolism involves the NADH/NAD couple (where NAD^+^ is the nicotinamide adenine dinucleotide that can be reduced to NADH via dehydrogenases), which is ubiquitous in living organisms. Other molecules are used as an active element in pharmaceutic drugs. In all of these cases, the reactions that are responsible for their activity involve an exchange of a proton H^+^ and electron. However, the reactions are not very sensitive to the cation, and therefore present an opportunity to explore organic electrodes with H^+^ that is substituted for metal cation: Li^+^, Na^+^, K^+^, and Mg^2+^. A good example was recently reported [9], which is discussed below. However, neither K^+^-ion nor Mg^2+^-ion batteries are mature. The state-of-the art on these batteries was reviewed through the point-of-view of their salts and solvents [10]. Attention in the present work is more focused on Li- and Na-ion organic electrodes, which were recently the subject of extensive research, since the progress on their electrochemical properties now suggests that they are promising for the development of a new generation of Li- and Na-ion batteries. Owing to the low sensitivity to the cation size, the same molecules/backbones of organic molecules usually work equally well for Li and Na-ion batteries and they were investigated.

**Figure 1:**
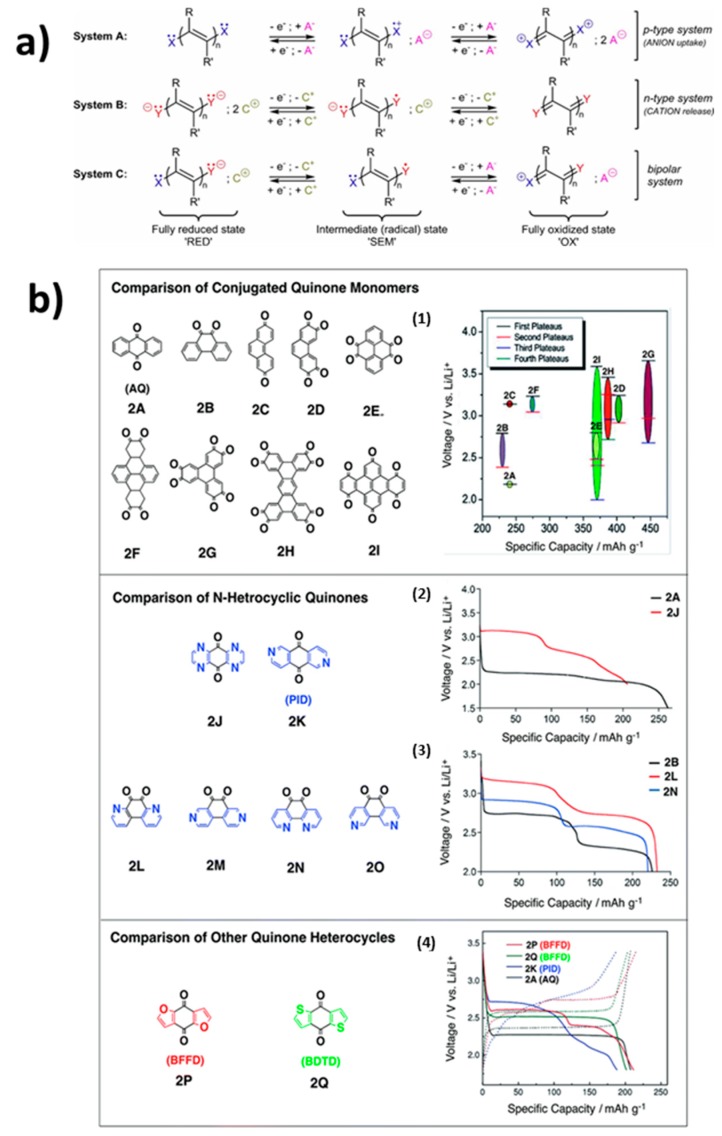
(**a**) General key redox-active organic systems and their related charge transfer steps. X/Y could be N, O, S, P, π-systems but also carboxylate, anhydride, or amide functional groups; R, R′ being potentiality integrated within the same cyclic structure (Reprinted with permission from [12]; copyright 2014 American Chemical Society. (**b**) Structures and electrochemical properties of (**b1**) selected quinone derivatives with varying degrees of aromatic conjugation; (**b2**) N-heterocycle derivatives of 9,10-anthraquinone and phenanthrenequinone; and, (**b3**,**b4**) other quinone heterocycles in comparison with their non-heterocyclic parent compounds. BFFD = benzofuro[5,6-b]furan-4,8-dione, BDTD = benzo[1,2-b:4,5-b’]dithiophene-4,8-dione (reprinted with permission from [52]; copyright 2016 Royal Society of Chemistry).

The redox-active organic molecules and their charge transfer steps adapted from Hünig’s classification [11] are reproduced in Figure 1a [12]. In this figure, X/Y are N, O, S, P, π-systems, but also carboxylate, anhydride, or amide functional groups. R, R′ can be integrated within the same cyclic structure. The results that are published in the literature [13,14,15,16,17,18,19] show that system B appears as the most relevant for developing organic Li-ion batteries with cations C^+^.

As an example, among the molecules with oxygen as the end group (Y = O in Figure 1a), dilithium (2,5-dilithium-oxy)-terephthalate (denoted Li4-p-DHT) demonstrated good electrochemical properties with fast kinetics and good cyclability [20]. However, the problem is to find lithiated enolate structures (‘RED’ form of the Syst. B) that are electrochemically active at a sufficiently high potential [21,22,23]. An elegant example of a strategy to increase the operating potential was found by switching from the para- to ortho-position in the quinone/hydroquinone moiety. In particular, dilithium (2,3-dilithium-oxy)-terephthalate (denoted Li4-o-DHT), which is simply the ortho-regioisomer of Li4-p-DHT, operated at an average potential of 2.85 V against 2.55 V with Li4-p-DHT [12]. However, the capacity, 105 mAh g^−1^ at 0.2C using 1 mol L^−1^ LiPF_6_ in ethylene carbonate (EC) and dimethyl carbonate (DMC) (1:1 by *v*/*v*) as electrolyte, was half that expected from the two-electron reaction for the corresponding quinonic form. Reducing the particle size was needed to obtain the full capacity of Li4-p-DHT [24], and the same should hold true for Li4-o-DHT.

Several difficulties limited the development of organic electrodes, despite the advantages that we have just recalled. First, the organic molecules have a propensity to dissolve into the electrolytes, which results in a poor cycle and calendar life. A battery without significant fade after more than 1100 cycles (three years, if charged once per day) is a basic requirement for many applications, and organic-based electrodes have difficulties to meet this requirement when compared to the state-of-the art inorganic electrodes. Intensive research then focused on the solution to this problem. Different methods were proposed to limit the dissolution of the organic electrodes in the electrolytes: including multiple (≥2) negative charges that result in salts of high lattice energy instead of neutral molecular structures [17], or immobilize the soluble organic materials onto solid substrates [25]. However, grafting on a solid substrate is detrimental to the specific capacity of the cells.

Another strategy is to increase the molecular weight by polymerization [26,27,28]. However, again, increasing the weight reduces the energy density so that a compromise has to be found between the cycle ability and energy density. Redox polymers are conducting polymers, nitroxyl radical polymers, carbonyl polymers, and sulfur containing polymers [29]. They have many advantages, such as film forming ability, flexibility, abundant resources, versatile chemical structures, tunable redox properties, and recyclability [30,31]. They are attractive for different applications in a number of electrochemical devices, such as batteries, biosensors, electrochromic devices, or biofuel cells [32]. Among them, carbonyl-containing polymers, such as polyimides and polyquinones, are the most promising as electrodes for the next generation of rechargeable batteries, because they have the best electrochemical properties [33]. For instance, quinone-based polymers were constructed by linking the monomeric units with various organic groups, such as methylene (–CH_2_–) [34] (see below, the section regarding conjugated compounds with n-quinone units), imine (–NH–) [35], ether (–O–), and thioether groups (–S–) [36,37,38], or just grafted on a skeleton chain [39,40]. Among the polymers in which the oxidized states are stabilized through π–π interactions between redox-active groups, poly(3-vinyl-*N*-methylphenothiazine) showed excellent cycling stability in a cell with LiPF_6_ in EC:DMC (1:1 by *v*/*v*) as electrolyte (after 10,000 cycles at a 10C rate, 93% of the initial capacity was retained), in addition to a high rate capability due to supramolecular hole transport [41]. However, the capacity was very modest (50 mAh g^−1^).

Organic carbonyl compounds have a high theoretical capacity, but in many cases only half (or less) of the carbonyl sites are electrochemically active [17,42,43,44] because the densely incorporated carbonyl groups usually lead to low active site utilization. Additionally, the highest average potential that was obtained by carbonyl-based cells to date is 2.8 V [25]. According to the functional structures that are used to stabilize the negatively charged carbonyl groups, the carbonyl electrode compounds are categorized into three types. Type I compounds employ a neighboring carbonyl to form stable enolates, for example, tetraketopiperazine [43]. In type II compounds, the carbonyl groups are directly connected to an aromatic core, which disperses charge by delocalization [13,42,44,45]. In type III compounds, the main stabilizing force comes from the formation of extra aromatic systems after electron injection [6,25,26,28,46,47]. This type III family contains two examples of four-Li uptake: C_6_O_6_Li_2_ and 5,7,12,14-pentacenetetrone, which emphasizes the important role of aromaticity. This guided Liang at al. [48] to start with extended conjugated systems, into which the pre-aromatic 1,2-dicarbonyl moieties are embedded, and arrange the carbonyl sites in such a configuration that adjacency (as in type I), direct connection to aromatics (as in type II), and the participation of enlarged conjugation upon reduction (as in type III) are simultaneously achieved. We can cite the symmetrical pyrene-4,5,9,10-tetraone (PTO) among such molecules that were built. The tetraone has the potential to undergo four reversible reductions, leading to a tetra-anionic structure for each pyrene, and the binding of four Li^+^ ions leads to a theoretical capacity of 408 mAh g^−1^. In practice, it undergoes a little less than four-electron reduction at an average potential of 2.59 V, as it reaches a reversible capacity of 360 mAh g^−1^ at 0.1C (1 mol L^−1^ LiPF_6_ in EC:DMC electrolyte), but the tetraone itself is slightly soluble, despite π stacking [48]. However, a polymer to which the PTO units are bound to the backbone of the polymeric structure of poly(methyacryloyl chloride) by the amide linkage delivered a capacity of 231 mAh g^−1^ with 83% capacity being retained after 500 cycles at 1C rate, and a very good charge-discharge ability (90% of the capacity at 30C as compared to 1C) in the LiNTf_2_/tetraglyme ionic-liquid system [39]. In this structure, the capacity is close to the theoretical capacity of the polymer, which is 262 mAh g^−1^. The 1,2-diketones are excellent lithium coordination sites. Liang et al. determined that the large negative free energy change from the delithiated state to the tetralithiated state was the reason why all four carbonyl groups are involved in the reversible reaction of PTO from DFT calculations, while this was found to be impossible in the case of other molecules that also contain four carbonyl groups, like *N*,*N*’-diphenyl-2,3,5,6-tetraketopiperazine and 7,1,4,5,8-naphthalenetetracarboxylic dianhydride. Only two carbonyl groups out of four participated in the electrochemical process of *N*,*N*’-diphenyl-1,4,5,8-naphthalenetetracarboxylic diimide [49].

In comparison with PTO, 1,10-phenanthroline-5,6-dione (PhenQ) delivered a smaller capacity, 231 mAh g^−1^ (0.2C, 1 mol L^−1^ LiPF_6_ in EC:DMC electrolyte), but the average potential was higher (2.74 V) [48]. After a drop during the first 10 cycles, the capacity that is delivered by PTO stabilizes and reaches 250 mAh g^−1^ after 50 cycles. Note, however, that 20 wt.% carbon was added to the PTO, which limits the capacity of the whole cell. This is a recurrent problem with organic-based electrodes, where a large amount of carbon, usually more than 30 wt.%, is blended with active materials to increase the conductivity [50]. The high capacity of PTO is ranked second to that of C_6_O_6_Li_2_ (580 mAh g^−1^ at 0.1C in the same electrolyte at a lower average potential of 2.2 V) [6]. The authors [48] attributed the successful four-Li uptake capability of C_6_O_6_Li_2_ to the fact that C_6_O^4−^ shares the character of type III molecules, which form an additional aromatic system (benzenoid in this case) when reduced to promote stabilization. This matches with the computation of the structural properties of lithiated oxocarbon salt (Li_2+*x*_C_6_O_6_) by using Perdew–Burke–Ernzerhof (PBE) exchange–correlation parameterization (spin-polarized Generalized Gradient Approximation) of DFT, which adds low-gradient pair-wise dispersion potential to the conventional Kohn–Sham DFT Hamiltonian to take the van der Waals interaction among the molecules into account [51]. The computations accounted for the very large theoretical capacity of 580 mAh g^−1^, from *x* = 0 to *x* = 4. According to the computation, each Li is coordinated to four oxygen ions of two or four neighboring C_6_O_6_ units. However, most of carbonyl-based molecules have two-electron uptake, like quinones, which are the subject of the next section [52].

A second difficulty with organic materials is their poor conductivity. Therefore, the active organic material is mixed with some form of conductive carbon, which is usually in large amounts, up to 60 wt.%. The specific capacity is reported in the literature per gram of active product. Therefore, even though this capacity is much larger than the result that was obtained with inorganic electrodes, the capacity per gram of electrode, which is what matters for industrial applications, is not so impressive. To make a comparison with the performance that was obtained with inorganic electrodes, the amount of carbon that is added to the active inorganic active material in commercial LIBs is only typically 10 wt.%. That is why, for the most promising results on organic active materials, the amount of carbon that is used to prepare the electrode is significant to compare with the state-of-the-art LIBs with inorganic electrodes can be done. However, the low percentage carbon in inorganic materials is the result of extensive optimization and it can be hoped that organics can be also optimized, but probably with still higher carbon content.

The third limitation is the operational voltage, commonly being lower than 4 V for organic cathodes, which also limits the energy density. On a general basis, sulfur-based compounds, such as disulfides and thioethers, result in voltages below ≈2.0 V vs. Li^+^/Li and can, in some cases, be considered as anode materials, while nitrogen and free-radical molecules lead to higher voltage and are thus considered for cathodes. Efforts to increase the working potential for cathodes or decrease it for anodes consist in adjusting the structure of the molecules.

Different classes of organic molecules were proposed through the years: quinones [13,23,25,34,36,37,48,50,52], imides [17,19,43,53], nitroxides [27,54], disulfides [55,56], carboxylates, [13,50], carbonyl compounds [7,17,25,26,42,57], as well as trioxotriangulene [58], antiaromatic corroles [59], triazines [60], and tetracyano quino-di-methane [61]. A table of 120 organic materials with their electrochemical properties as the active elements of electrodes for Li/Na batteries is available [62].

Data on many molecules are available in many reviews [21,22,32,52,54,55,56,57,58,59,60,61,62,63,64,65,66], which cover the historical developments and introduction on electrochemical energy storage. The purpose of the present work is to report the advances that have been obtained. Therefore, we have selected the best results that were obtained to overcome the limitations mentioned above and to discuss the most promising routes to obtain organic electrodes that are competitive with state-of-the-art inorganic-based LIBs and SIBs.

## 2. Quinones

Quinones are a class of organic compounds that are derived from aromatic compounds by the conversion of an even number of –CH= groups into –C(=O)– groups, resulting in a fully conjugated cyclic dione structure. For example, o- and p-benzoquinones (C_6_H_4_O_2_) are the archetypal members of quinones that are derived from the benzene aromatic group, and 1,4-benzoquinone (BQ), simply called “quinone”, is the name of this class. The two other important members of this family are naphtoquinone and anthraquinone C_14_H_8_O_2_ (AQ, 2A in Figure 1b), for which the parent aromatic hydrocarbons are naphtalene and anthracene, respectively. In nature, quinones play a vital role in numerous electrochemical reactions for energy transduction and storage, including respiration and photosynthesis. Recent studies provide evidence for quinone’s utilities in energy applications through the hybridization of quinone molecules with various organic and inorganic materials [67,68,69,70,71]. Advances in the design of quinone-functionalized hybrid materials for different applications, such as artificial photosynthetic platforms, phototransistors, plasmonic light harvesting platforms, and dye-sensitized solar cells, are reviewed elsewhere [72]. They were also studied as pseudocapacitors [73,74,75,76].

The redox properties of simple quinone systems, such as p-benzoquinone (parabenzoquinone, 1,2 dione) or o-benzoquinone (orthobenzoquinone, 1,4 dione) (BQ), were thoroughly studied a long time ago, both experimentally [77,78] and computationally [79,80]. In practice, the advantage of BQ is the increase of operational potential when switching from the para to the ortho-position of the quinone. The two plateaus in voltage-capacity curves in Figure 1b evidence the two consecutive one-electron steps involving the redox activity of quinone in organic electrolytes. The exchange of two electrons and two Li^+^ ions combined with the low molar mass of quinones results in high theoretical charge capacities up to 500 mAh g^−1^ [33], so their energy density is comparable to those of inorganic cathode materials for Li-ion batteries. It is then not surprising that the reversible redox couples of quinones have been applied to Li batteries [23,36,37,48,81,82,83].

The triptycene molecule bearing three BQ units in a rigid tripod structure exhibits five-electron redox reactions, which practically provide a specific capacity as high as 387 mAh g^−1^ in Li-ion coin cells at 0.1C in a solution containing 2 mol L^−1^ LiN(CF_3_-SO_2_)_2_ in a (1:1 by *v*/*v*) mixture of dimethoxyethane (DME) and 1,3-dioxolane (DOL) with 1 wt.% LiNO_3_ as an electrolyte. However, the capacity decreased to 210 mAh g^−1^ after only 20 cycles [84]. This provides evidence that the benzoquinones, and actually pristine quinone-based molecules, dissolve in non-aqueous electrolytes unless they are already charged, like in Li_2_C_6_O_6_.

### 2.1. Adjustment of the Redox Potential

The redox potential (1.8–3 V) is low. To compete with the inorganic-based cathodes in terms of energy density, the potential must be increased. According to Clar’s theory, the electrons transferred at the carbonyl groups of quinones upon lithiation or sodiation can delocalize over the aromatic carbon skeleton of the whole molecule. Therefore, the composition of this carbon skeleton impacts lithiation voltages. In particular, Wu et al. [85] examined the correlation between the electron delocalization (aromaticity) and the lithiation voltage of the carbonyl-containing polycyclic aromatic hydrocarbons by density functional theory (DFT) computations. The results that are illustrated in Figure 1b were well explained by Clar’s aromatic sextet theory. The computations demonstrated that: (i) increasing the amount of carbonyl functional groups at optimal positions on the polycyclic aromatic hydrocarbons infrastructures increases the aromaticity difference between the products and the reactants of lithiation, which subsequently increases the voltage; (ii) employing full Clar polycyclic aromatic hydrocarbons (largest number of disjoint aromatic π-sextets or their derived structures as parent molecules), ensures high specific capacity. As an illustration of these results, the compound 2G in the figure displayed both the highest voltage and specific capacity of all the molecules that were studied in [85] due to its highly conjugated carbon skeleton with multiple rings and the increased number of carbonyl groups. The redox potential is also improved by the introduction of electron-withdrawing groups [86], like carboxylic groups. It is also possible to increase the number of bound Li atoms in some quinones (e.g., anthraquinone-2-carboxylic acid and anthraquinone-2,6-dicarboxylic acid), which is further discussed in the section devoted to carboxylates. Density-functional calculations predicted the formal potentials of the carbonyl-based organic molecules [87]. The highest formal potentials of 2.7−3.2 V vs. Li^+^/Li were obtained when sulfur was used as the heteroatom, as compared to 2.3−3.2 V in the case of oxygen heteroatom. The lowest potential is obtained with NH-containing molecules, because they have the lowest unoccupied molecular orbital (LUMO) energy level. It is harder to reduce the molecules with higher LUMO energy values. The effect of the position of the carbonyl groups, and the result from low to high potential are in the sequence 3 < 2 < 1 < 4 < 5 < 6. The authors explained this sequence by the fact that the largest Li^+^ binding energies are obtained when the carbonyls are in the ortho positions (organic motifs 4, 5, and 6), which is also consistent with the fact that these motifs have the lowest LUMO energy levels. To further increase the potential, electron-withdrawing R groups, in particular, R=NO_2_ can be used; in which case, the potential increases to 3.9 V vs. Li^+^/Li. However, the energy density is limited because of the increase in the molar mass. Another strategy can be used to avoid this mass penalty. In particular, adding more electronegative atoms to the ring can modify the molecular motif. In particular, computations have shown that an increase of energy density is predicted when substituting carbon with nitrogen. This substitution should also result in higher aromaticity and stability.

**Figure 2:**
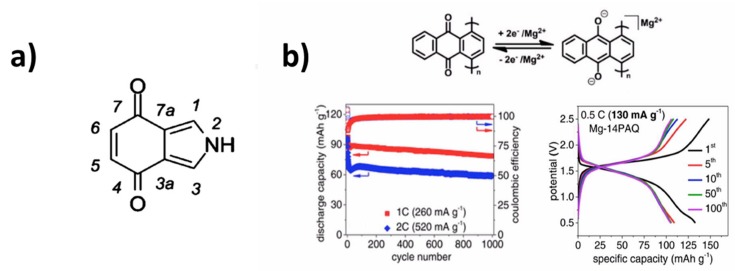
(**a**) Structure of isoindole-4,7-dione with numbering of the ring atoms (Reproduced with permission from [88]; copyright 2012 American Chemical Society) and, (**b**) Long-term cycling and representative charge-discharge galvanostatic curves for 1,4-polyanthraquinone P14AQ as a cathode of Mg-battery with 0.3 m magnesium bis(hexamethyldisilazide) Mg(HMDS)_2_-4MgCl_2_/tetrahydrofuran (THF) as the electrolyte (Reproduced with permission from [89]; copyright 2016 Wiley).

The ring fusion of pyrrole and p -benzoquinone results in isoindole-4,7-dione (IID) (see Figure 2a) and is a derivative of both pyrrole and quinone compounds that were investigated for use as electrode material in lithium-ion batteries (LIBs) [7,25,26]. The corresponding dilithium salt has a theoretical capacity of 333 mAh g^−1^. The redox potentials of the quinone moiety in the IID derivatives are lower than those of other quinone derivatives, because of the electron-donating effect of the fused pyrrole ring. As a consequence, the calculated redox potentials of most compounds in the IID series is low, typically 2 V vs. Li^+^/Li. Nevertheless, because of the much higher theoretical capacity, the theoretical specific energy is comparable to that of inorganic cathode materials. However, a few compounds are oxidized at higher potentials, which are identified by DFT calculations in [88] and reported in Table 1, due to the electron-withdrawing effect of their substituents.

These three IID derivatives have a higher theoretical specific energy than LIBs. The third derivative in the table also has additional reductions at lower potentials, providing protection against overreduction. However, the IID molecules are soluble in organic solvents. A common strategy to solve this problem is to increase the molecular weight by polymerization [26,27,28]. Isoindoles can be polymerized on the 1 and 3 positions [90]. In the present case, the pyrrole moiety in IIDs offers the possibility to polymerize these compounds by redox coupling, resulting in a conducting polymer with a polypyrrole backbone. The polymerizing IID produces polymers similar to that made in Ref. [28], in which a polyaniline derivative that is fused with p-benzoquinone moieties was investigated. In the corresponding polymer, the quinone redox reaction occurs at a potential where the polypyrrole backbone is oxidized and conductive, which should increase the rate capability.

The dissolution of the quinone-based molecules in the organic electrolytes is due to their hydrophobicity and low molecular weights. Thus, it is an intrinsic property and has been experienced in Li- and Na-ion batteries, but also when used as organic electrodes for Mg-batteries. In this context, the 2,5-dimethoxy-1,4-benzoquinone (DMBQ) is the most promising, as it operates at a potential above 2.0 V vs. Mg^2+^/Mg, however, the capacity dropped fast after 30 cycles [91].

### 2.2. Reduction of the Solubility

All of the neutral quinones dissolve in the electrolytes, which prevents their direct use as electrodes.

#### 2.2.1. Benzoquinones

The solubility of 1,4-benzoquinone that is functionalized with two methoxy groups is lowered due to intramolecular forces (e.g., π–π interaction and hydrogen bonding) [46]. Another strategy to solve the dissolution problem is the attachment of quinone molecules to carbon-based conducting nanomaterials (e.g., carbon nanotubes (CNTs) and graphene) through π–π interaction [92] covalent anchoring [25,93], the use of solid electrolytes [82], or quinone-based polymers [94].

Lithium batteries using p- and o-quinones with two lithiooxycarbonyl (–CO_2_Li) groups (e.g., 9,10-anthraquinone, 9,10-phenanthrenequinone, and pyrene-4,5,9,10-tetraone) as cathode materials exhibit excellent cyclability when compared to their parent quinones. In particular, pyrene-4,5,9,10-tetraone having two –CO_2_Li as a cathode delivered an initial capacity of 217 mAh g^−1^ at 0.2C rate in a cell with 1 mol L^−1^ LiPF_6_ in propylene carbonate (PC) as electrolyte, which is 73% of the theoretical capacity. The capacity retention was 86% after 20 cycles, which was mainly due to the drop of capacity in the first five cycles, and the mean discharge voltage was 2.39 V vs. Li^+^/Li [95]. Unfortunately, more cycles are needed to test the stability of the electrode and the ability of the lithiooxycarbonyl groups to mitigate the dissolution of the active species in the electrolyte.

Kim et al. proposed a high-energy organic cathode using a quinone-derivative, tetrachloro-1,4-benzoquinone (C_6_C_14_O_2_), for use in sodium-ion batteries [96]. The authors followed the strategy that consists in tuning the working potential by modifying the elemental species in the quinone derivatives while maintaining the electroactivity of the C=O redox centers to increase the energy density. Benzoquinones bearing perfluoroalkyl groups already raised the voltage to 3.1 V vs. Li^+^/Li in LIBs [83]. Unfortunately, the increased molecular weight due to the long alkyl chains decreased the specific capacity, so that the energy density was reduced. To circumvent this effect, Kim et al. used a substitution with halogen atoms, such as fluorine and chlorine, to increase the redox potential of the benzoquinone derivatives. The high electronegativity of the halogen groups draws electrons from the molecule, thereby increasing the redox potential of the C=O redox centers without a crippling increase of the molecular weight. The results were in accordance with the expectation, but only for one cycle, as the capacity dropped very fast due to the dissolution of the organic molecule in the electrolyte. 

Jing et al. proposed two oligomeric hetero-aromatic-fused quinones, namely BDTD (poly(benzo(1,2-b:4,5-b’)dithiophene-4,8-dione-2,6-diyl)) and PBDTDS (poly(benzo(1,2-b:4,5-b’)dithiophene-4,8-dione-2,6-diylsulfide). Actually, these are cross-conjugated oligomeric quinones, since they possess three unsaturated groups, of which two are conjugated to a third, but they are not conjugated to each other [97]. The insertion of sulfur atoms between the BDTD units modifies the molecular conformation from planar in PBDTD to helical in PBDTDS. These quinones were tested in cells with a polypropylene membrane that was used as a separator, and 1 mol L^−1^ LiClO_4_ in dioxolane-dimethoxyethane (1:1 by *v*/*v*) as the electrolyte. Each cathode contained 66 wt.% of composites consisting in one of the two quinones plus 16 wt.% of carbon nanotubes. Both of the quinones delivered comparable capacity over 200 mAh g^−1^ at 2.5 V vs. Li^+^/Li with 96% retention over 250 cycles. However, PBDTD showed superior rate capability, which was attributed to its planar conformation, which favors π–π stacking and hence inter-chain electron transport. On the other hand, 4,8-dihydrobenzo(1,2-b:4,5-b0)dithiophene-4,8-dione (BDT) being used as a cathode for SIB showed poor cycle life, unless it is associated to graphene, in which case the composite delivered a capacity of 217 mAh g^−1^ at 0.1C. The capacity decreased during the ten first cycles, but it was then stable at 175 mAh g^−1^ over 70 cycles in cathodes containing 40 wt.% acetylene black [98].

Song et al. [99] proposed a polymer cathode material poly(benzoquinonyl sulfide) (PBQS), linking benzoquinone units with thioether bonds. At current density of 50 mA g^−1^, they showed a high energy density of 734 Wh kg^−1^ (2.67 V × 275 mAh g^−1^) in Li batteries that were tested with 1 mol L^−1^ lithium bis(trifluoromethane-sulfonyl)imide (LiTFSI) in a mixed solvent system of 1,3-dioxolane (DOL) and 1,2-dimethoxyethane (DME) as the electrolyte, or 557 Wh kg^−1^ (2.08 V × 268 mAh g^−1^) in Na batteries that were tested with 1 mol L^−1^ NaTFSI/DOL + DME electrolyte [38]. These high capacities show that the benzoquinone units in PBQS are fully utilized. In Li batteries, the average discharge voltage was 2.20 V, even at the high current rate of 5000 mA g^−1^ (thus the power density is 11 kW kg^−1^), releasing a large capacity of 198 mAh g^−1^ in less than 2.5 min. for an electrode with active material loading of 60% (1–2 mg cm^−2^). This rate capability was attributable to the fast redox kinetics of the quinone groups [37]. At a current rate of 500 mA g^−1^, the capacity still remained 86% at the 1000th cycle, which demonstrated that the link between benzoquinone units with thioether efficiently prevented dissolution in the electrolyte. On another hand, the rate capability and cyclability were not satisfactory in Na batteries. This is a situation that is often met with redox active polymers: the polymerization effectively mitigates the dissolution of the active material in the electrolyte, but the rate capability is reduced because the polymer is not a good conductor.

Senoh et al. proposed an original approach [100]. Since BQ dissolves into the electrolyte, the authors modified the design of the cell accordingly. On the cathode side, 2,5-dipropoxy-1,4-benzoquinone (DPBQ) was used as redox couples that dissolved in a liquid electrolyte that was separated by a solid electrolyte diaphragm (a plate of lithium ion-conducting glass–ceramic), which prevented the dissolved active materials from reaching the counter-electrode (Li foil). Under such conditions, a discharge capacity of 5 μmol DPBQ in 1 mol L^−1^ LiClO_4_/GBL at current 56.5 μA was maintained constant at 233 mAh g^−1^ over 25 cycles. Even though this is an attractive result, it should be noted that this is a capacity per gram of active product DPBQ that is in very low concentration in the electrolyte, so that the capacity per gram of cathode will be very small.

Dimerization is an interesting approach, since it does not reduce the capacity. The capacity of 2,2’-bis-p-benzoquinone (BBQ) was significantly larger than that of single BQ derivatives [101]. The BBQ showed a discharge voltage of 2.9 V vs. Li^+^/Li and a discharge capacity of 170 mAh g^−1^ (52% of the initial capacity) after 20 cycles in a cell with 1 mol L^−1^ LiPF_6_ in EC (30 vol%) and diethyl carbonate (DEC) (70 vol%) electrolyte. The cyclability is better than that of BQ, which only maintains 30% of its capacity after 20 cycles, but still the problem of dissolution in the electrolyte has not been permanently solved by dimerization.

Luo et al. studied 2,3,5,6-tetraphthalimido-1,4-benzoquinone (TPB), in which rigid groups coordinate to a molecular benzoquinone skeleton. It contains four rigid aromatic phthalimide groups that are directly linked to the benzoquinone framework at symmetrical positions and contains 10 active carbonyl sites [102]. The four rigid aromatic groups lower the energy of the lowest unoccupied molecular orbital (LUMO), so that the voltage discharge potential is raised to 3.05 V for the first plateau (the second plateau is at 2.0 V). As an organic electrode (50 wt.% TPB, and 40 wt.% carbon black (Super P^TM^), plus 10 wt.% polyvinylidene fluoride (PVdF)) for LIB with an aprotic 1 mol L^−1^ LiTFSI/DOL + DME electrolyte in which TPB is insoluble, the initial discharge capacity was 223 mAh g^−1^ at 0.2C, with 91.4% capacity retention after 100 cycles, and the rate capability was high (155 mAh g^−1^ at 10C). The capacity per gram of cathode is less attractive, but this is a very good result in an organic electrode. Furthermore, the enhanced structural conjugation has efficiently suppressed the dissolution of the organic material in the electrolyte (LiTFSI in dioxolane/dimethoxyethane).

#### 2.2.2. Anthraquinones

Like in the case of benzoquinone, the first approach towards increasing the working potential of anthraquinone consisted of adding a functional group to the anthracene molecule. The effect of substituting the electron donating and electron withdrawing groups on redox windows, and solvation free energies was computed by the DFT approach for ∼50 anthraquinone (AQ) derivatives [103]. In particular, sodium functional groups (–SO_3_Na) modified anthraquinone were investigated as cathodes for Li batteries. The Na_2_C_14_H_6_O_8_S_2_ with two –SO_3_Na showed the best performance [104]. The working potential was raised to 2.4 V, owing to the electron withdrawing character of the sulfonic group. When combined with graphene paper, the capacity was maintained at 125 mAh g^−1^ after 100 cycles at 0.1C, against 135 mAh g^−1^ after 20 cycles. Therefore, the capacity is not large, mainly because SO_3_Na– is electrochemically inactive during the electrochemical process and it adds weight, but the cyclability was improved. Moreover, the rate capability was good, but only after combination with graphene paper.

The AQ derivatives with more hydroxyl groups exhibit higher discharge voltage with better cycle performance. This general trend is exemplified in [105] by comparison of the electrochemical properties of 1,5-dihydroanthraquinone (1,5-DHAQ), 1,2,7-trihydroanthraquinone (1,2,7-THAQ), and 1,2,5,8-tetrahydroanthraquinone (1,2,5,8-THAQ). The increased size of the molecules with the number of hydroxyl groups creating H-bonds reduces the solubility in the electrolyte, and thus improves cycle life. The DFT calculations show that the negative charge increases with an increasing number of OH^−^ group, with two beneficial effects: (i) it facilitates the lithiation and thus improves the rate capability; and, (ii) n addition, it increases the reduction potential (3.0 V for 1,2,7-HAQ and 1,2,5,8-THAQ against ~2 V in AQ). The most successful approach to improving the electrochemical properties is polymerization and, as mentioned in the introduction, the most promising redox polymers belong to the class of carbonyl-containing polymers. Among them, polyanthraquinone (PAQ) and poly(anthraquinonyl sulfide) (PAQS) are the most popular, which combine the good reversibility of the anthraquinone moiety with the stability and poor solubility of aromatic polymers.

Poly(1,4-anthraquinone) (P14AQ) performed better than poly(1,5-anthraquinone) as a cathode for Li-ion batteries [106]. At 0.2C, with 1 mol L^−1^ LiTFSI in DOL + DME (2:1 by *v*/*v*) electrolyte, the capacity was close to theoretical (263 mAh g^−1^), with a capacity retention of 98.3% after 100 cycles. The average discharge voltage was 2.14 V, so the energy density was calculated to be 560 Wh kg^−1^ (2.14 V × 263 mAh g^−1^), which is actually slightly higher than that of LiCoO_2_ (530 Wh kg^−1^). The rate capability was also very good; even at 20C, the capacity was maintained at 182 mAh g^−1^ and the average discharge voltage was 1.97 V, so that the energy density of 360 Wh kg^−1^ was attained in 2 min. In addition, P14AQ demonstrated a very small voltage gap between the charge and discharge curves (2.18 − 2.14 = 0.04 V), 99.4% capacity retention after 1000 cycles, and a drop to 69% of the low-rate capacity when the discharge was reduced to just 2 min. Most importantly, the cycling stability is one of the highest among the organic electrodes, showing that the dissolution in the electrolyte (which prevents the use of anthraquinone alone as an electrode for instance) was completely suppressed in this polymer. However, these results are, like usual, reported per gram of active material (P14AQ), and the authors failed to provide the exact composition of the cathode. Therefore, direct comparison with inorganic electrodes remains difficult, because the composition per gram of the cathode is missing, as well as the amount of carbon in wt.% in the organic cathodes, usually much larger than in inorganic ones.

Poly(anthraquinonyl sulfide) (PAQS) was evaluated as an electrode material to increase the operating potential. The delocalization of electrons over the entire molecule is assumed to enhance the redox peak shift to more positive potential in PAQS than that of anthraquinone due to the enlarged π-system. A PAQS electrode shows the reduction plateau at an average voltage of 2.2 V and the oxidation plateau at 2.3 V, which is small when compared with the typical redox potential of benzoquinone (2.8 V) [34,99]. The electrochemical performance of PAQS also depends on the chemical structure. The effect was investigated on two PAQS polymers: poly(1,5-anthraquinonyl sulfide), in which the sulfur connections are at the 1 and 5 positions of the anthraquinonyl structure (abbreviated as P15AQS) and poly(1,8-anthraquinonyl sulfide), in which the sulfur connections are at the 1 and 8 positions (i.e., P18AQS) [107]. The performance of these two anthraquinone-based organic cathode materials significantly depends on the substitution positions, as determined by their starting dichloroanthraquinone chemicals (15DCAQ and 18DCAQ) used during the synthesis process. In 1,5-dichloroanthraquinone 15DCAQ, the two Cl atoms are well separated on two phenyl rings that are oriented in diametrically opposite directions. During its condensation polymerization with Na_2_S, the anthraquinonyl groups are chemically bonded through S atoms at the 1 or 5 substitution position of one anthraquinonyl group and the 1 or 5 position of another anthraquinoyl group. The polymer chain linearly grows in opposite directions. On the other hand, the polymer chain of P18AQS grows in a stacked and layered structure. The steric effect between the neighboring anthraquinonyl groups is large, which indicates that the reactions of condensation polymerization between 18DCAQ and Na_2_S can hardly proceed to a high degree. The consequence is that the capacity of P15AQS is larger than that of P18AQS at any rate. Therefore, only the P15AQS chemical structure is of interest for energy storage and it is referred simply as PAQS. In addition, Xu et al. have also demonstrated that the electrochemical performance also depends on the choice of the electrolyte and the binder. With 1 mol L^−1^ LiTFSI in 1,3-dioxolane (DOL):dimethoxyethane (DME) (1:1 by *w*/*w*) electrolyte, the capacity of up to 5C of P15AQS is larger with a PVDF binder than with Clevios^TM^ P (CP). Furthermore, the electrochemical performance is much better in this electrolyte than in 1 mol L^−1^ LiPF_6_ in EC/DMC. The poor cycle life of P15AQS in the carbonate-based electrolyte is attributed to the continuous irreversible reaction of the reduced anthraquinone structure with carbonate solvents via nucleophilic attack. Ether-based electrolytes are favorable to P15AQS, both for high capacity and good cycle life. Therefore, the electrochemical properties that are reported below on PAQS only consider P15AQS in ether-based electrolytes.

PAQS in 1 mol L^−1^ LiTFSI in DOL:DME as electrolyte delivered a stable capacity of 185 mAh g^−1^ over 200 cycles at 50 mA g^−1^. This good cycle ability demonstrated that polymers with thioether bonds (C–S–C) could effectively circumvent dissolution. Increasing the current density to 500 mA g^−1^ only reduced the capacity by 18% [36]. An all-organic Na-ion battery using p-dopable polytriphenylamine as cathode and n-type redox-active PAQS as anode demonstrated a specific energy of 92 Wh kg^−1^, with a voltage output of 1.8 V. A 60% capacity was obtained at the very high rate of 16C (3200 mA g^−1^) and 85% capacity retention was achieved after 500 cycles at 8C rate [108]. Furthermore, its relatively simple synthetic pathway allows for the chemical combination with nanomaterials such as graphene or carbon nanotubes [36,109]. For these reasons, PAQS was also successfully implemented as a cathode in several battery technologies, such as sodium, magnesium, and potassium batteries [89,99,110,111,112]. However, in the case of Mg-ion batteries, a comparative study of the electrochemical properties of P14AQ, P26AQ, and PAQS with the choice of 0.3 mol L^−1^ Mg(HMDS)_2_-4MgCl_2_ (HMDS: hexamethyldisilazide) in THF (tetrahydrofuran) electrolyte showed that P14AQ is the winner [89] (see Figure 2b). PAQS was not capable of providing sustainable and long-term cycling performance, despite the choice of this non-nucleophilic electrolyte. At contrast, P14AQ demonstrated a much better cycle ability. At 0.5C (130 mA g^−1^), an important capacity loss was still observed in the first seven cycles, from 132.7 to 106.0 mAh g^−1^, but then, the capacity loss of P14AQ was only 1% between the 7th and the 100th cycle, with a coulombic efficiency >99%. At 1C, the reversible capacity, 87.1 mAh g^−1^, at the 10th cycle was maintained at 83.7 and 78.7 mAh g^−1^ after 500 and 1000 cycles. 

The cycleability was attributed to the fact that the redox-active quinonyl moieties in 14PAQ lie on the side of the main polymer chain, which allows for the rotation of the anthraquinonyl groups along the polymer chain, inducing flexibility. This rotation flexibility helps to minimize the space hindrance and relaxes the structural stress of the polymer, which in turn provides better structure stability for P14AQ. Indeed, minimizing the hindrance plays a more important role than in Li-ion batteries, since magnesium, though the same size as Li^+^, is much more polarizing and it rigidifies the host polymer. In addition, the two adjacent carbonyl (C=O) groups via the chelating effect during discharge better stabilize the inserted magnesium cation. 

A similar positive chelating effect on the cycle ability was observed when a redox-active quinone-based organic polymer was used in Li-ion batteries [37]. The high-rate capability was attributed to the much smaller HOMO–LUMO gap in P14AQ when compared to other polyanthraquinonyl derivatives [106]. The stepwise two-electron redox potentials of P14AQ were experimentally measured at about 1.6 and 1.7 V vs. Mg^2+^/Mg.

Good results were also obtained with PAQS in SIBs. An all- organic Na-ion battery using the n-type redox-active PAQS anode and p-dopable polytriphenylamine (PTPAn) cathode yielded a specific energy of 92 Wh kg^−1^ and an average operating discharge voltage of 1.8 V [108]. The electrolyte was a mixed DOL/DME solution that was saturated with NaPF_6_. During discharge, the Na^+^ cations and PF^−^ anions de-inserted the anodic PAQS and cathodic PTPAn chains, respectively. As an anode-limited design, the reversible capacity of the cell was determined by the mass of anode material and it reached 220 mAh g^−1^. A high-rate capability was demonstrated, with a reversible capacity of 130 and 118 mAh g^−1^ at 16C and 32C, respectively, with 85% capacity retention and 99% coulombic efficiency over 500 cycles at 8C rate. It is possible to increase the capacity of PAQS by enriching it with sulfur. Sulfur-enriched PAQ*_x_*S polymers with different polysulfide segment lengths (*x* between 2 and 9 sulfur atoms) were synthesized in high yields by an in-situ reaction to form sodium polysulfides with 1,5-dichloroanthraquinone [113]. The lithium coin cell tests of the PAQ_x_S redox polymer cathodes indicated higher steady-state capacity (>225 mAh g^−1^) than PAQS.

The use of quinones as high-performance anode materials for aqueous rechargeable batteries recently showed remarkable results [9] (see Table 2) in cells that are based on the selected quinone anode PAQS, pyrene-4,5,9,10-tetraone (PTO), and a polymerized version of PTO, namely PPTO. High anode specific capacity (200–395 mAh g^−1^), fast kinetics (67–84% charge/discharge capacity at 10C), and state-of-the-art specific energy/energy densities (up to 76–92 Wh kg^−1^ /161–208 Wh L^−1^) for several pH conditions (−1 to 15), charge carrier species (H^+^, Li^+^, Na^+^, K^+^, Mg^2+^), temperature (−35 to 25 °C), and atmosphere (with/without O_2_), and long cycle life (3000 cycles/3500 h) were demonstrated. A PAQS anode in an alkaline battery (electrolyte with pH>14 in conjunction with highly industrially mature NiO_2_H_x_/cathode) delivered a capacity of 200 mAh g^−1^ at 0.2C and 180 mAh g^−1^ at 1C (200 mA g^−1^), with 88% capacity retention after 1350 cycles at 1C at 100% depth of discharge. These results show the promises of quinone redox chemistry. In particular, the quinones might solve the problem of lack of anodes that, so far, prevented the use of aqueous batteries for large-scale energy storage applications.

#### 2.2.3. Naphtoquinones Derivatives

**Figure 3:**
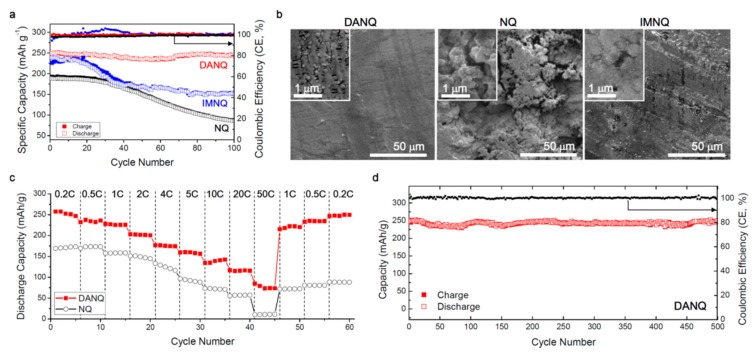
(**a**) Discharge/charge capacities and coulombic efficiencies of the Li//2,3-diamino-1,4-naphthoquinone (DANQ) and Li//1*H*-naphtho[2,3-*d*]imidazole-4,9-dione (IMNQ) cells, compared to those of Li-naphthoquinone (NQ) cell for 100 cycles at 0.2C rate. (**b**) Surface morphologies of the separators of each cell taken after 50 cycles by SEM. (**c**) Rate performance of the Li-DANQ cell, as compared to that of the Li-NQ cell. (**d**) Discharge/charge capacities and coulombic efficiencies of the Li-DANQ cell at 0.2C with an extended life of 500 cycles (Reproduced with permission from [114]; copyright 2016 American Chemical Society).

A remarkable result with NQ-derivative was obtained by adding amino groups at the 2- and 3-positions of the NQ ring to form 2,3-diamino-1,4-naphthoquinone (DANQ) [114]. This material was tested in an electrode that was prepared by integrating DANQ, Super P carbon, and polyvinylidene (PVdF) binder in a weight ratio of 60:30:10. The cell contained a Li anode, in 1 mol L^−1^ LiTFSI in a mixture of 1,3-dioxolane and dimethoxy-ethane in volume ratio of 2:1. The two-step reduction of the carbonyl groups in the enolates, followed by oxidation of the enolate groups, was observed at 2.33 and 2.17 V (lithiation) and 2.41 and 2.27 V (delithiation). The initial capacity at 0.2C rate was 250 mAh g^−1^ (363 mAh cm^−3^), with a capacity retention of 98% after 100 cycles and coulombic efficiency >99% during cycling in the potential range 1.8–3.0 V. The rate capability was remarkable at 117 mAh g^−1^ (170 mAh cm^−3^) and was still retained at 20C. This result is attributable to the fact that DANQ is well crystallized and is a semiconductor with relatively small gap of 2.7 V, which increases the conductivity and the lithium diffusion coefficient in the range 1–6 × 10^−7^ cm^2^ s^−1^. The crucial preparation of the cell to obtain good cycle life involved the use of a porous gas diffusion layer (GDL) as the current collector. The GDL surfaces were chemically modified with carboxylic acid groups (GDL−COOH) to induce the formation of peptide bonds with the amino groups of DANQ. The Li-DANQ cell demonstrated a capacity of 248 mAh g^−1^ (360 mAh cm^−3^) after 500 cycles at 0.2C, owing to this bonding, which amounts to a capacity retention of 99% cell and coulombic efficiency >99% throughout (see Figure 3). This is one of the best results in terms of rate capability and cyclability for an organic electrode. The energy density of the Li-DANQ cell reached 575 Wh kg^−1^, and a high value of 300 Wh kg^−1^ was maintained at a high-power density of 10,000 W kg^−1^, which was even better than the results that were obtained on quinone/carbon composites [115]. The addition of amino groups was very effective to stabilize organic molecules and was not limited to naphthoquinones. Their presence in 2,3-diamino-phenazine (DAP) also suppressed dissolution in the solution of DOL:DME (1:1 by *v*/*v*) that was used in the electrolyte, and consequently enhanced its electrochemical performance [116]. At a current density of 0.1 A g^−1^, the discharge capacity of DAP was 220 mAh g^−1^, comprising 86% of the initial capacity after 100 cycles. The capacity at 1 A g^−1^ was still 90 mAh g^−1^. This functionalization by amino groups is promising to stabilize the active molecules in organic electrodes.

### 2.3. Conjugated Compounds with n Quinone Units

The limitation of utilizing active sites, such as high concentrations of carbonyl groups, was discussed earlier. This guided Huang et al. to use macrocyclic molecules with several p-quinone units, because the macrocyclic structure increases the efficiency of the use of active sites. A cell with calix [4] quinone cathode, and poly(methacrylate) (PMA)/poly-(ethylene glycol) (PEG)-based gel polymer electrolyte (GPE) hybrid loading with 0.7 mol L^−1^ LiClO_4_ in dimethyl sulfoxide and Li-anode delivered a capacity of 379 mAh g^−1^ after 100 cycles at 0.2C [34]. However, the conventional liquid electrolyte components in this gel polymer are believed to be harmful for battery performance [117]. This led Zhu et al. to replace the gel-polymer with the composite polymer electrolyte poly(methacrylate) (PMA)/poly(ethylene glycol) (PEG)-LiClO_4_-3 wt.% SiO_2_, which has an optimum ionic conductivity of 0.26 mS cm^−1^ at room temperature [82]. 

The cathode was another macrocyclic molecule, pillar[5]quinone (P5Q), containing five quinone units that are linked by methylene bridges at para positions [118,119,120], with a pillar architecture that is favorable to Li uptake. The pillar[5]quinone cathode in this all-solid-state battery with Li-anode and poly(methacrylate) (PMA)/poly(ethylene glycol) (PEG) electrolyte showed an average voltage of ∼2.6 V and a high initial capacity of 418 mAh g^−1^, with 94.7% capacity retention after 50 cycles at 0.2C rate (89.2 mA g^−1^) through the reversible redox reactions of enolate/quinonid carbonyl groups. The capacities that were obtained at 0.3, 0.5, and 1C were 361, 286, and 197 mAh g^−1^ in the first cycle, but continuously decreased upon cycling at 0.55C and were even faster at 1C. These measurements show that the rate capability is limited, but the performance at 0.2C was remarkable, with a capacity that compares well with the theoretical value of 446 mAh g^−1^, corresponding to 10 Li uptake by the five quinone units. This result demonstrates the promising prospect of macrocyclic molecules for device application. This performance is also attributable to the choice of the polymer electrolyte, since organic cathode compounds that are deemed unusable in liquid electrolyte may turn usable in solid-state lithium cells. Hexa-aza-trinaphthylene and hexa-aza-triphenylene-hexacarbonitrile are other examples [121].

### 2.4. Carboxyl Based Materials

3,4,9,10-perylenetetracarboxylicdianhydride (PTCDA) is an aromatic carbonyl compound that has an aromatic core and two anhydride groups (C_24_H_8_O_6_). PTCDA is reduced in two-electrons processes until each carbonyl group accepts one electron together with one cation to form an enolate during the discharging process. However, in a potassium-ion battery with 0.5 mol L^−1^ KPF_6_ in EC:DEC (1:1 by volume) as the electrolyte, the PTCDA electrode exhibits a capacity of 131 mAh g^−1^ in the potential range 1.5–3.5 V vs. K^+^/K, with a capacity retention of 66.1% over 200 cycles at the current density 50 mAh g^−1^ [122]. This capacity corresponds to the insertion of only two K^+^ ions in this potential range that react with the carbonyl groups in PTCDA to form potassium enolates [42,44]. When PTCDA was discharged down to 0.01 V, PTCDA delivered a capacity of 753 mAh g^−1^, but only during the first cycle; after 30 cycles, the excess capacity with respect to the result in the potential range of 1.5–3.5 V vanished. PTCDA was also studied as a cathode for Li-ion and Na-ion batteries [18,123,124]. The results indicate that the cycling performance was not good in LIBs, but was promising in Na-ion batteries. A very high capacity at low voltage, but only in the first cycles, was observed for PTCDA in Na-ion batteries, as well as with other homologs, such as 1,4,5,8-naphthalenetetracarboxylic dianhydride (NTCDA) [125].

Fédèle et al. improved their π-extended naphthyl-based dicarboxylate electrode [16] by replacing the central core a 4,4′-biphenyl unit leading to the dilithium 4,4′-biphenyldicarboxylate (Li_2_−BPDC) to increase the separation distance of the two end-carboxylate units when compared to the closely related 2,6-naphthalene and 1,4-phenyl counterparts [126]. The Li_2_−BPDC material has been tested in a cell with 1 mol L^−1^ LiPF_6_ in EC:DMC (1:1 by *v*/*v*) as the electrolyte. After the formation of the SEI, i.e. at the second cycle, the Li_2_−BPDC delivered a capacity of 220 mAh g^−1^ at a cycling rate of 0.2 e^−^/hour and maintained a capacity of 182 mAh g^−1^ (86.4% of theoretical capacity) after 25 cycles at 2 e^−^/hour (1C rate). This enhanced power rate of the material is due to the additive effects of π-conjugation and the increased distance between inorganic layers. The size of the organic spacer, which modifies the distance between the electro-active carboxylate units, plays a noticeable role in improving the electrode performance by increasing lithium-ion conduction in the structure. However, the capacity retention is still limited, as the capacity decreased by 20 mAh g^−1^ over 24 cycles. Prior results that were obtained on the sodium counterpart, Na_2_−BPDC, showed that Na_2_−BPDC is a promising anode for sodium-ion batteries. Tested in a cell with 0.8 mol L^−1^ NaClO_4_ in EC:DEC (1:1 by *v*/*v*) as the electrolyte, in an electrode that contained 57.1 wt.% of active product, Na_2_−BPDC delivered a reversible capacity of about 200 mAh g^−1^ at ca. 0.5 V vs. Na^+^/Na., the cell exhibited stable cycle performance over 150 cycles, and excellent rate performance of 100 mAh g^−1^, even at a 20C rate [127]. However, this result was due to the fact that different crystal structures of Na_2_−BPDC with different degrees of deprotonation of the carboxylic acid (COOH) groups are obtained, depending on the preparation process, and only the fully deprotonated Na_2_−BPDC showed the promising results that are mentioned above.

Croconic acid disodium salt (CADS) has a cyclopentene backbone with five carbonyl groups, which gives rises to a conjugated structure, and can thus participate in the lithiation reaction. Luo et al. synthesized CADS nanowires (150 nm in diameter) for cathodes in lithium batteries by anti-solvent crystallization [128]. The cathode in a LIB with 1 mol L^−1^ LiPF_6_ in EC:DEC (1:1 by *v*/*v*) as the electrolyte delivered a reversible capability of 177 mAh g^−1^ at a current density of 0.2C, and it retained 170 mAh g^−1^ after 110 cycles. In addition, 50% of the 0.1C capacity was still retained when the current density increases to 6C. This improvement with respect to bulk CADS particles is due to the small diffusion length of the nanosized wires, which improves the capacity and rate capability, and also facilitates the accommodation of CADS to the change of volume during cycling to improve cycle life. More importantly, these results were obtained with a cathode that contained only a few wt.% carbon. This result illustrates that a controlled nanostructure leads to greatly improved electrochemical reactions. Another example is the morphological control and size effect of sodium rhodizonate (SR), a carbonyl-based organic salt Na_2_C_6_O_6_, when used as a cathode in rechargeable SIBs [129] with 1 mol L^−1^ NaClO_4_ in in a mixture of ethylene carbonate/propylene carbonate (EC:PC, 1:1 by volume) with 5% fluoroethylene carbonate (FEC) as electrolyte. The SR nanorods with uniform diameters of ca. 200 nm exhibited the best sodium-ion storage properties, 190 mAh g^−1^ at 0.1C and they retained 90% of its second cycle capacity after 100 cycles. The choice of the binder is an important element that contributes to the performance of Na_2_C_6_O_6_. An electrode containing SR, conductive carbon black (super-P), and carboxymethyl cellulose (CMC) as binder (weight ratio: 70:20:10) in water was compared with the same electrode with the traditional PVdF binder [130]. When cycled between 3.0 and 1.0 V at a rate of 50 mA g^−1^, the cell capacity with the CMC binder was 173.5 mAh g^−1^ after the second cycle, retaining 147.5 mAh g^−1^ after 100 cycles, i.e., 80% capacity retention when compared to 36% after 40 cycles in the cell with PVdF binder. Remarkably, the electrodes using CMC binder showed a capacity as high as 116 mAh g^−1^, with 93% capacity retention at 1000 mA g^−1^. Surprisingly, the capacity retention is thus much better at 1000 mA g^−1^ than at 50 mA g^−1^. Therefore, battery degradation is not due to the number of cycles, but rather is due to the time during which the cell is cycling (the time spent to achieve 1000 cycles at 1000 mA g^−1^ is the same as the time spent to perform 50 cycles at 50 mA g^−1^). In other words, the problem with the cell containing the CMC binder is calendar life and not cycle life, which suggests that dissolution of the material in the electrolyte was not involved. This result also contradicts the claim by Lee at al. that the irreversible phase transformation of Na_2_C_6_O_6_ during cycling is the origin of the deteriorating redox activity [131]. Wang et al. also claimed that the flake morphology facilitates ionic diffusion and thus increases the rate capability. Although the morphology is an important parameter, the nanosize of the active material is the crucial parameter, as shown by the results in [128]. In the same way, disodium terephthalate, Na_2_C_8_H_4_O_4_, with nanosheet-like morphology in a cell where the electrolyte was 1 mol L^−1^ NaClO_4_ in a mixture of EC:DEC (1:1 by volume), exhibited considerably improved electrochemical properties—higher reversible capacity (248 vs. 199 mAh g^−1^), higher rate capabilities (for instance, 1.55 times that of the bulk material at 1250 mA g^−1^), and better cycling performance (105 vs. 60 mAh g^−1^ after 100 cycles at 250 mA g^−1^) when compared with the bulk material [132].

Carboxylic acids and esters contain carbonyl groups with a second oxygen atom that is bonded to the carbon atom in the carbonyl group by a single bond. The conjugated dicarboxylates e.g., dilithium terephthalate (Li_2_C_8_H_4_O_4_), dilithium trans-trans-muconate (Li_2_C_6_H_4_O_4_), and dilithium 2,6-naphthalene dicarboxylate (2,6-Naph(COOLi)_2_) are potential anodes for LIBs [13,133]. The tests were made in coin cells with 1 mol L^−1^ LiPF_6_ that was dissolved in a solution of ethylene carbonate, dimethyl carbonate, and ethyl methyl carbonate (30:40:30 volume ratio, respectively) as the electrolyte. At 0.05C, the porous microspheres consisting of lithium terephthalate, Li_2_C_8_H_4_O_4_, nanoparticles coated uniformly with nitrogen-doped carbon delivered a capacity of 221 mAh g^−1^, and 68% capacity retention after 50 cycles [134]. Note that this capacity is calculated per gram of active material, but the electrode only contained 60 wt.% of it, since 30 wt.% conductive carbon and 10 wt.% binder were added. Most of all, a low Li^+^ ion insertion voltage at 0.8 V vs. Li^+^/Li and a high reversible capacity of 301 mAh g^−1^ were demonstrated at 80 °C with a poly(ethylene oxide) (PEO)-based solid-state electrolyte [13].

Organic sodium-ion electrodes that are composed of oxygen-containing functional groups (carboxylate and carbonyl groups) [53,135] are limited by the fact that these functional groups only store one sodium ion reversibly per C=O unit. Doping sulfur into the organic electrodes potentially enhances the conductivity and increases the amount of stored sodium ions [136]. Zhao et al. replaced O atoms by sulfur in the sodium salt of terephthalate (PTA-Na) based on these considerations and molecular engineering techniques [137]. When four sulfur atoms are introduced, the capacity increased to 567 mAh g^−1^ at a current density of 50 mA g^−1^ when using 60 μL 1 mol L^−1^ NaClO_4_ in EC/DMC (1:1 by *v*/*v*) with 5% FEC as electrolyte, but the capacity retention at 500 mA g^−1^ after 200 cycles was only 60%.

Wang et al. [138] used the organic tetrasodium salt of 2,5-dihydroxyterephthalic acid (Na_4_DHTPA; Na_4_C_8_H_2_O_6_) as the initial active material for both the electrodes of a sodium-ion battery, with 1 mol L^−1^ NaClO_4_ in EC:DMC (1:1 by *v*/*v*) electrolyte. The reversible uptake/removal of two Na^+^ ions is associated with the enolate groups at 1.6–2.8 V (Na_2_C_8_H_2_O_6_/Na_4_C_8_H_2_O_6_, positive electrode) and the carboxylate groups at 0.1–1.8 V (Na_4_C_8_H_2_O_6_/Na_6_C_8_H_2_O_6_, negative electrode). This was the first example of all-organic rocking-chair SIBs, maintaining an average voltage of 1.8 V and a practical energy density of about 65 Wh kg^−1^. However, 25% of the capacity was lost after 100 cycles at 19 mAh g^−1^.

In terephthalate (TP) salts, the exchange of metallic cation can reduce dissolution into liquid electrolytes. In particular, substitution of Li for Ca significantly improved the electrochemical properties [139,140]. With 1 mol L^−1^ LiPF_6_ solution in a mixture of EC:DMC (1:1 by *v*/*v*) as the electrolyte (impossible with PEO, which dissolves into this standard electrolyte), calcium terephthalate CaC_8_H_4_O_4_ delivered a capacity of 399 mAh g^−1^ at 0.1C, without any capacity decay in the voltage window 0.005–3.0 V. Other TP salts, including *M*C_8_H_4_O_4_, have also been tested as anodes for LIBs, with *M* = Sr and Ba [141] and compared with CaTP. In this work, the best results were obtained with CaTP, which has the best cycling performance with the highest discharge capacity, from 170 mAh g^−1^ at the 5th cycle to 155 mAh g^−1^ at the 50th cycle with a capacity fade of 0.19% per cycle. However, this capacity retention is still not very good, and the solubility in EC-DMC solvents after one month at 45 °C is 13.3 mg L^−1^. The CaTP sample has a pair of redox peaks at 0.64 and 1.24 V, which indicates that different metallic cations can affect the voltage, in a way that is similar to the induction effect in inorganic electrodes. This low voltage vs. Li^+^/Li was attributed to the existence of the stronger electrostatic interaction between Ca^+^ and –COO^−^, as it is more difficult to break the C=O bond and regroup the electrons into a set of new p-bonds. XRD experiments confirmed this. The Ca^2+^ cation with smaller ionic radius has a stronger interaction with the carboxylate group, which resulted in the electron being localized in two unidentical bonds of –COO groups (C=O double bond length was 1.19 Å and C–O single bond length was 1.38 Å). The stronger interaction between the substituted metal atom and oxygen atom in the COO group is observed by FTIR experiments [141], and it should provide improved stability of the terephthalate. Other examples illustrating the use of the isomeric effect to modify the electronic structure to improve the electrochemical properties of organic cathode materials are the voltage gain in lithiated enolate-based materials [12] and the introduction of two lithio-oxycarbonyl groups to enhance cycle life [95]. Recently, easily synthesized silver terephthalate Ag_2_TP nanoparticles gave much better results than other TP salts [142]. The Ag_2_TP anode exhibited an average discharge capacity of 149 mAh g^−1^ at 0.5C for 500 cycles and nearly 100% coulombic efficiency, and 133 mAh g^−1^ at 1C for 100 cycles, in a Li-ion battery with 1 mol L^−1^ LiPF_6_ containing a three-component solution of EC:DEC:DMC with a 1:1:1 volume ratio. The Ag_2_TP anode in Na-ion battery with 1 mol L^−1^ NaClO_4_ with EC:DMC (1:1 by *v*/*v*) electrolyte displayed 133 mAh g^−1^ at 1C for 100 cycles.

Disodium terephthalate, Na_2_C_8_H_4_O_4_, has been recognized as a promising anode material for SIBs [136,143], with reported Na storage capacities of up to two Na atoms per molecule (corresponding to about 255 mAh g^−1^). The inserted Na atoms prefer to bind at carboxylate sites up to one Na atom per molecule. However, the hexagonal sites (on the aromatic ring) become dominant according to DFT computations for higher Na concentrations [144]. Chen et al. used DFT to compare sodium attachment to disodium terephthalate (Na_2_TP) and a related molecule disodium pyridine dicarboxylate (Na_2_PDC). As a result, the substitution of the Na_2_TP’s aromatic ring with pyridine will lead to an increased voltage by about 0.4 V vs. Na_2+*x*_TP up to Na_2+1_PDC and a similar voltage to the terephthalate between Na_2+1_PDC and Na_2+2_PDC, i.e., a two-plateau behavior vs. a single plateau for Na_2+*x*_TP [145].

**Figure 4:**
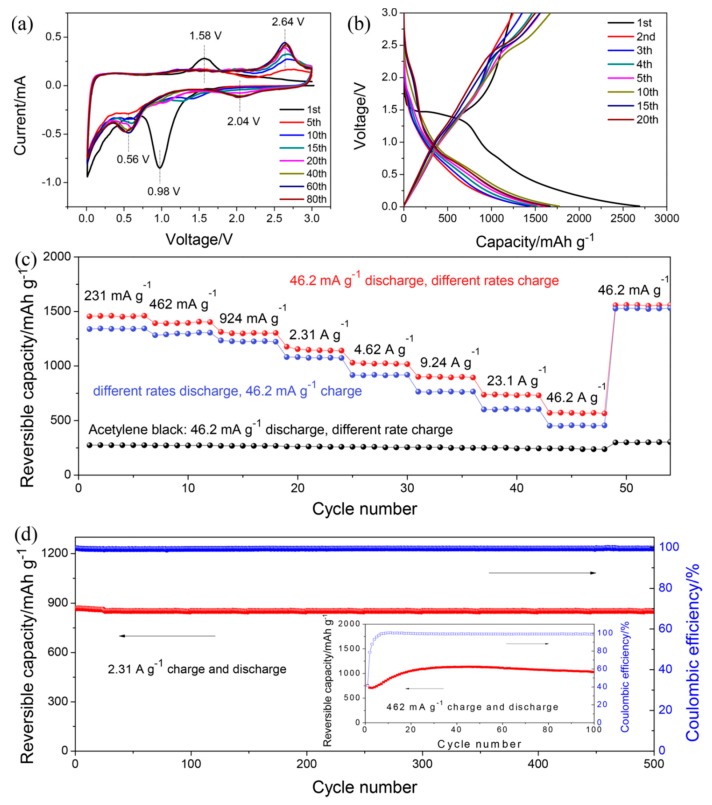
Electrochemical performances of the maleic acid anode in the voltage range of 0.01–3.0 V. (**a**) Cyclic voltammograms; (**b**) charge and discharge profiles at 46.2 mA g^−1^; (**c**) rate capability at room temperature after 20 cycles at 46.2 mA g^−1^ (red and blue symbols indicate charge and discharge rate capability, respectively); and, (**d**) cycling and coulombic efficiency at current density of 2.31 A g^−1^ after rate test (the inset is the cycling and coulombic efficiency at current density of 462 mA g^−1^ from initial cycles) (Reproduced with permission from [146]; copyright 2017 American Chemical Society).

A remarkable result was obtained with maleic acid as an organic anode, owing to its small volume effect and unique Li-ion storage mechanism, divided in five steps, as illustrated in Figure 4 [146]. The electrolyte was 1 mol L^−1^ LiPF_6_ in EC:DEC:DMC (1:1:1 by *v*/*v*). First, two carboxyl hydrogen atoms composed of maleic acid are irreversibly replaced by two lithium ions. Subsequently, a two-electron reduction reaction occurs on the two carbonyl groups. Afterwards, another two-electron reduction occurs. In the fourth step, two-electron reduction continues on the two carbonyl groups. The last step is another two-electron reduction. In all, eight electrons are involved in this scheme, while the experimental value of the capacity, 1500 mAh g^−1^ at 46.2 mA g^−1^, corresponds to 12 electrons that are involved in the reduction process. The difference was attributed to the surface adsorption of lithium. The rate capability is also very good: at 4.62, 23.1, and 46.2 A g^−1^ discharge rate, the electrode is still able to deliver 919.6, 607.2, and 457.2 mA g^−1^, respectively. At 2.31 A g^−1^ current density, a capacity retention of 98.1% is obtained after 500 electrochemical cycles, which demonstrates the superior cycling stability of the carboxyl-based anode.

### 2.5. Modifications by Cyanide Functional Groups

Replacing the oxygen atoms of 1,4 benzoquinone by two cyanides (–C≡N) provides 7,7,8,8-tetracyano-*p*-quinodimethane, or simply tetracyanoquinodimethane (TCNQ). Hayu and Honma proposed a solid-state lithium cell using a TCNQ cathode [61]. The three-layer solid electrolyte was composed of 20-mm thick PEO layer on the cathode side, 400-mm thick 1-ethyl-3-methyl-imidazolium bis(trifluoromethylsulfonyl)imide ([EMIm][Tf2N]) ionic liquid (IL)-silica ‘‘saggy sand’’ layer in the middle, and a controlled SEI on the anode side. The PEO film is soluble in most ILs and, upon cell assembly, the PEO layer transforms into a highly viscous thin matrix of PEO-IL-silica mixture that bonded the solid electrolyte to the cathode and prevented dissolution. The third SEI layer on the anode side was prepared by applying a small amount (5 ml) of EC:DEC on the anode surface before cell assembly to modify the surface with desirable Li-conductive decomposition products. The Li cell, with a cathode containing 50 wt.% TCNQ, reached a capacity of 216 mAh per g_TCNQ_ at room temperature and 0.2C discharge rate, but it decreased to 170 mAh per g_TCNQ_ after 100 cycles. In addition, the voltage plateaus at about 3.2 and 2.5 V are too low to achieve high energy density when used as a cathode and too high for use as an anode. According to computational studies, higher voltages are expected to be obtained by changing from TCNQ to tetracyanoethylene (TCNE) [147,148,149]. The lithiation (sodiation) of TCNQ only involves the coordination of Li (Na) and CN, while the aromatic ring is inactive. TCNE has the advantage of eliminating the inactive aromatic ring, and the molecule is smaller. With the molecular TCNE crystal, the specific capacities that are computed within a dispersion-corrected density functional theory (DFT) are 1247 mAh g^−1^ for Li and 416 mAh g^−1^ for Na, which correspond to volumetric capacities of 1845 and 615 mAh cm^−3^ for Li and Na, respectively. The computed voltage can reach 3.54 V vs. Li^+^/Li for Li insertion and 3.31 V vs. Na^+^/Na for Na insertion, so that this material could be cathode-active.

Banda et al. found that cyanide substitution on perylene diimide raises the reduction potentials and voltage profiles of perylene diimide-based organic electrodes for SIBs because cyanide is a good electron-withdrawing group. The authors in this work also determined that a twist into the perylene ring could also increase the potential. However, the potential could only increase to 2.6 V [150]. An increased redox potential by cyanide substitution was also observed in other organic electrodes, such as hexaazatriphenylen hexacarbonitrile HAT-CN(6) [123]. In the same way, the substitution of an electron-donating group, or electron-donating atom, such as a halogen, which is intended to increase the conductivity and thus the rate capability, reduced the redox potential, which is an undesired effect for a cathode. This was observed, for example, by substituting methyl group and fluorine to 7,7,8,8-tetracyanodimethoquinone (TCNQ) [151].

### 2.6. Quinone Based Flow Batteries

Quinone was also considered in redox flow batteries [152,153,154,155]. In contrast to Li-ion batteries operating in organic (or solid) electrolytes, aqueous redox flow batteries store electrical energy via reversible electrochemical reactions between redox-active aqueous electrolytes and cell electrodes, and they are competitive with other energy storage devices for large-scale application [156]. In this context, the quinone/hydroquinone redox couples are attractive due to their high kinetics of the charge transfer and their high solubility in aqueous solvent (>1 mol L^−1^ in a pH 0 electrolyte for 9,10-anthraquinone-2,7-disulphonic acid (AQDS) [157,158]. Anthraquinone (AQ) derivatives are suitable redox components for the anode in redox flow batteries because of their low reduction potential [159]. A quinone–bromide acidic redox flow battery (QBFB) that contained an AQDS anolyte and bromide catholyte in H_2_SO_4_ (1 mol L^−1^) working at 40 °C showed a peak galvanic power density of 0.6 W cm^−2^ at 1.3 A cm^−2^ [157]. The chemical modification of the AQDS backbone with the hydroxyl group improved the solubility of AQDS in an aqueous medium and the cell voltage. Hydroxylated AQ [e.g., 2,6-dihydroxyanthraquinone (2,6-DHAQ)] was used in an alkaline (1 mol L^−1^ KOH) redox flow battery, where ferrocyanide replaced the toxic bromide [160]. The open-circuit voltage increased by 47% when compared to the acidic QBFB case, with an equilibrium potential of 1.2 V vs. ferri/ferrocyanide. Further modification of 2,6-DHAQ to 2,3,6,7-tetrahydroxyquinone or 1,5-dimethyl-2,6-DHAQ raised the potential to 1.35 V. A power density that was higher than 0.45 W cm^−2^ at room temperature was obtained, with 99% retention over 100 cycles at 0.1 A cm^−2^.

The compounds, 1,2-benzoquinone (BQ) and 2,3-naphthoquinone (NQ), can be used in the catholyte of redox flow batteries due to their high standard electrode potential E_0_ > 0.9 V [161], which can be further increased by functionalization with electron-withdrawing groups (e.g., –CHO, –CN, –COOH, –COOCH_3_, and –NO_2_). A redox flow battery with 1,2-BQ-3,5-disulfonic acid (*E*_0_ = 0.85 V) catholyte and AQ-2-sulfonic acid anolyte achieved an equilibrium potential of 0.94 V [161].

Organic materials, such as quinones, which were already mentioned, as well as quinoxaline [162] and thiophene [163] in the anolytes, can be coupled with a 4 V (or higher) catholyte to form an all-organic redox flow battery. For instance, the reduction potential of anthraquinone is 2.2 V vs Li^+^/Li, which can be combined with 2,5-di-tert-butyl-1,4-bis(2-methoxyethoxy)benzene [164] (oxidation potential of 4 V) to form a redox couple of 1.8 V.

### 2.7. Quinone Based Supercapacitors

The fast kinetics of the redox reaction of quinones make them attractive in pseudocapacitor electrodes and to combine with carbon to produce an electrode with enhanced capacitance by adding their complementary contributions in an electrical double-layer capacitor (EDLC). Hierarchical porous carbon nanotubes (HPCNTs) that were decorated with AQ molecules showed a specific capacitance of 710 F g^−1^ at 1 A g^−1^ in 1 mol L^−1^ H_2_SO_4_ at the optimum mass ratio AQ:HPCNT of 7:5, and the capacitance retention after 1000 cycles at 10 A g^−1^ was 96.45% [165]. At 1 A g^−1^, the specific capacitance of pristine HPCNTs and pure AQ was only 304 F g^−1^ and 42 F g^−1^, which were far below the capacitance of AQ-HPCNT. Thus, this result gives evidence of the strong synergistic effects between AQ and HPCNTs. Pyrene-4,5 dione, a quinone with chemical formula C_16_H_8_O_2_, attached to carbon onions increased the capacitance to 130 F g^−1^ in 1 mol L^−1^ H_2_SO_4_ at the scan rate of 5 mV s^−1^, and 97% capacitance retention after 10 000 charge/discharge cycles at 1.3 A g^−1^ [73]. The polydopamine (PDA)-decorated graphene oxide/poly(3,4-ethylenedioxythiophene) (GO/PEDOT) hybrid showed a capacitance of 126 F g^−1^ at 1 A g^−1^ in 0.1 mol L^−1^ LiClO_4_ [166]. Polynorepinephrine-functionalized graphene oxide sponge showed a capacitance of 232 F g^−1^ at 1 A g^−1^ in 1 mol L^−1^ H_2_SO_4_ [167]. 2,6-dibromoanthraquinone, polymerized on a Ketjenblack carbon electrode to avoid the dissolution of quinone in the organic electrolyte, had a capacitance of 650 F g^−1^ at a scan rate of 5 mV s^−1^ in a 0.5 mol L^−1^ LiClO_4_/acetonitrile mixture, with 85% capacitance retention after 1000 cycles [168]. An asymmetric capacitor with anthraquinone-modified carbon fabric as a negative electrode and ruthenium oxide as a positive electrode showed an energy density of 12.7 Wh kg^−1^ at 0.8 A discharge rate and an average power density of 17.3 kW kg^−1^ in 1 mol L^−1^ H_2_SO_4_ [169]. The operating voltage with the AQ-modified carbon electrode increased to 1.3 V when compared to 1.0 V in the symmetric RuO_2_-based capacitor. Note that the organic electrolyte, in this case, is important, because the voltage with an aqueous electrolyte is limited to 1.0 V due to water splitting. (PAQS)/graphene sheets (GSs) composite synthesized by in-situ polymerization demonstrated a specific capacitance of 349 F g^−1^ (86 mAh g^−1^) at a current density of 500 mA g^−1^, and a capacitance of 305 F g^−1^ was maintained at the high current density of 5000 mA g^−1^ [110].

## 3. Imides-Dianhydrides

Rylene diimides, such as perylene diimide or naphthalene diimide, accept up to two electrons at room temperature (at ∼2.5 V vs. Li^+^/Li), so that they were investigated as promising electrode materials. Recently, Zhang et al. computed the working potential of these diimides (and other promising candidates for organic electrodes, such as AQ, tetrachlorobenzoquinone, indigo, sulfur-substituted indigo) with high accuracy (5%) [170]. Actually, among rylene imides, the LUMO energy decreases and the electron affinity increases along the sequence pyromellitic diimide → naphthalene diimide → perylene diimide [44]. The increased electron affinity implies an increase of the average discharge voltage, so that perylene diimide is attractive over the other rylene imides. A series of PDI polymers were synthesized by reacting perylene dianhydride with a variety of diamines [18]. Again, polymerization reduced dissolution and improved cyclability, but this was at the expense of the rate capability. Rylene did not deliver more than 50% of their theoretical capacity at high C-rate, even in the presence of functionalized graphene [99,171]. Hydrazine-reduced carboxylic acid containing perylene diimide (benzoic-PDI) was chosen by Bhosale et al. for different reasons. First, the carboxylic acid is hydrophilic, so the solubility of the product in organic electrolytes is reduced. Second, n-doping using hydrazine improved the conductivity and rate capability [172]. This reduced benzoic-PDI in a cell with 1 mol L^−1^ LiPF_6_ in EC:DEC (1:1 by *v*/*v*) as the electrolyte exhibited 100% coulombic efficiency, even at 20C. At 5C, 88% of the theoretical capacity (based on two-electron transfer in the potential window 1.5–4.5 V) was retained, even after 200 cycles. The battery also exhibited very high specific energy (213 Wh kg^−1^) and specific power (8548 W kg^−1^). Furthermore, PDI was more successful that the Diels–Alder extension of perylene imides at the lateral position, despite the extension of the π-system, The capacity measured in a cell with 1 mol L^−1^ LiTFSI in 1,3-dioxacyclopentane/1,2-dimethoxyethane (DOL:DME, 1:1 by *v*/*v*) as the electrolyte was limited to 92 mAh g^−1^ at 1C, and a capacity retention of 75 and 50% after 100 and 300 cycles, respectively, with an operating voltage window of 1.60–2.80 V vs. Li^+^/Li, bcecause the porous solid-state network was only accessible to 42.2% cell volume [173].

Perylene-3,4,9,10-tetracarboxylic acid diimides and their derivatives represent one of the most promising class of electron-accepting materials, due to their outstanding chemical and physical properties, including high electron mobility. Their rigid, fused aromatic core favors π–π intermolecular interactions that impart n-type semiconducting properties are useful for many applications [174]. A simple perylene diimide, 3,4,9,10-perylene-bis(dicarboximide) (PTCDI), was proposed as a cathode for Na-batteries [175]. This molecule demonstrated reversible insertion/extraction of 2 Na^+^ ions per molecular unit, with a capacity of 140 mAh g^−1^ at 10 mA g^−1^, 103 mAh g^−1^ at 600 mA g^−1^, and good cyclability with 90% capacity retention over 300 cycles, in a cell with 1 mol L^−1^ NaPF_6_ in a mixed solvent of EC, DEC, and propylene carbonate (PC) (EC:DEC:PC, 45:45:10 vol%) as electrolyte because of the high density of redox-active carbonyl groups in a stable π-conjugated structure.

Naphthalene diimide (NDI) derivatives also exhibit a large charge capacity (e.g., 1,4,5,8-naphthalenetetracarboxdiimide: 201 mAh g^−1^) as the cathode-active material in Li-ion batteries [44]. A pendant-type naphthalene diimide-based polymer bearing a N-methyl group (N-methyl-N0-(50-norbornene-20-methyl) naphthalene-1,4,5,8-tetracarboxylic diimide) delivered a capacity of 138 mAh g^−1^ at 5C, which suggested that almost all of the NDI moiety contributed to charge storage, and 96 mAh g^−1^ at 20C. Most of all, the capacity retention at 10C was 90% over 1000 cycles [176]. The electrochemical measurements were performed under nitrogen and with 0.1 mol L^−1^ tetrabutylammonium hexafluorophosphate in CH_2_Cl_2_ as the electrolyte. The pendant-type NDI polymers are thus very promising as active cathode elements for Li-ion batteries.

Schon et al. constructed a three-dimensional (3D) covalent organic framework using a trans-functionalized triptycene as one building block and perylene diimide as the linker [177]. With this electrode, a lithium-ion battery with 1 mol L^−1^ LiPF_6_ in EC:DMC (1:1 by *v*/*v*) as the electrolyte delivered a capacity of 75.9 mAh g^−1^ with a voltage of 2.5 V vs. Li^+^/Li, retaining 88.2% capacity after 200 cycles. Moreover, it only lost an additional 8% of capacity in 300 cycles from cycle 200 to cycle 500.

Polyimide (PI) is a promising polymer as a cathode due to its high theoretical capacity, mechanical strength, and stable cycling performance. Most of the organic electrodes do not work with aqueous electrolytes, but polyimide is an exception [33,44,178].

Two polyimides using the redox-active anthraquinone group as the connection unit were synthesized and characterized as cathodes for sodium secondary batteries. They provided capacities of 165 and 192 mAh g^−1^ at 50 mA g^−1^, and were stable over 150 cycles in an electrolyte of NaPF_6_ dissolved (saturated) in DME:DOL (1:1 by *v*/*v*) [179]. The specific capacities were calculated according to the net mass of polyimides in the cathode, which contained 40 wt.% polyimide, 40 wt.% conductive carbon, and 20 wt.% PVDF).

**Figure 5:**
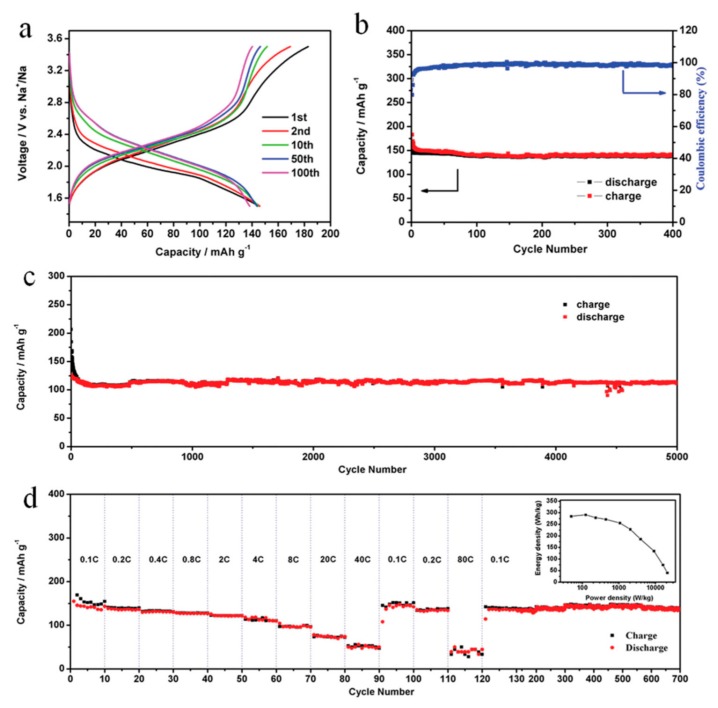
(**a**) Charge–discharge curves and (**b**) cycle performance of the dianhydride-based polyimide with 3,4,9,10-perylenetetracarboxylicdianhydride (PTCDA) as the active centers, and alkyl chain length C2 (PI2) electrode at a current density of 0.1 C obtained for a sodium-ion battery. (**c**) Cycle performance of the PI2 electrode at a current density of 0.8C. (**d**) Rate performance of the PI2 electrode at different current densities, inset: Ragone plots (Reproduced with permission from [180]; copyright 2014 Wiley).

A one-pot solution polymerization route prepared the PTCDA-based polyimides with PTCDA as the active centers, and alkyl chain length C2 to interconnect the PTCDA molecules. These electrodes in sodium batteries with 1 mol L^−1^ NaPF_6_ in EC:DMC (1:1 by *w*/*w*) as the electrolyte delivered a high specific power of 20.99 kW kg^−1^, specific energy of 285 Wh kg^−1^, and reversible capacity of 110.8 mAh g^−1^, even after 5000 cycles at 0.8C (87.5% of its initial capacity) [180] (see Figure 5). This is the best result that is obtained among a series of dianhydride-based polyimides that were investigated in this work, because of its low solubility and the lowest unoccupied molecular orbital (LUMO) energy. This remarkable result illustrates the efficiency of the insertion of small imide molecules in an organic polymer and it shows that the interconnecting alkyl chains are very efficient to prevent the dissolution of the product in the electrolyte by the perylene ring and the conjugated structure with the carbonyl groups. However, these optimized results were only obtained when a large amount of carbon was added to the polymer in the electrode, which reduced the polymer loading to 30%. This is another illustration of the recurrent use of excess amounts of carbon to increase the capacity with organic electrodes [23,39,48,181]. On the other hand, these results provide evidence that dianhydride-based polymer materials can make a stable and flexible framework. Actually, even commercial PTCDA sub-micrometer rods without any modification exhibit high electrochemical reversibility (≈140 mAh g^−1^ at 10 mA g^−1^), good rate performance (91 mAh g^−1^ at 1000 mA g^−1^), and good cycle life for 200 cycles in the potential range of 1–3 V in cells with 1 mol L^−1^ NaPF_6_ in EC:DEC (1:1 by *v*/*v*) [123].

A redox-active and water-insoluble polyimide, poly-(naphthalene four formyl ethylenediamine) (PNFE), undergoes a reversible redox reaction along with Na^+^ insertion–extraction in an aqueous electrolyte at low potential (0.50 V vs. Ag/AgCl) [182]. The PNFE anode in 1 mol L^−1^ Na_2_SO_4_ delivered a capacity of 134 mAh g^−1^ at 1C (100 mA g^−1^) and 112 mAh g^−1^ at 12C. In addition, the cyclability was remarkable, as no capacity fade was detected over 1000 cycles at 10C. An all- organic Na-ion cell using a poly(2,2,6,6-tetramethylpiperidinyloxy-4-yl vinylether) (PTVE) cathode, PNFE anode, and an aqueous Na_2_SO_4_ electrolyte yielded the high open circuit voltage of 1.75 V and delivered a reversible capacity of 75 mAh g^−1^ in terms of cathode capacity.

A polycondensation reaction of stoichiometric quantities of diamine PEO and dianhydride (pyromellitic-dianhydride (PMDA) and naphtalene-1,4,5,8-naphthalenetetracarboxylic dianhydride (NTDA)) synthesized polyimides–polyether multi-block copolymers [183]. The naphthalene polyimide with the longest PEO chain (PEO2000) gave the best result. The initial capacity was 300 mAh g^−1^, which is commonly observed with such organic cathodes when the cut-off voltage during cycling is as low as 1 V. After 10 cycles at 0.1C, the capacity decreased to 170 mAh g^−1^ (84% theoretical capacity) and was then almost constant up to the 40th cycle (i.e., the last cycle explored). The cathode was separated from the Li anode by a glass fiber that was imbibed with 1 mol L^−1^ LiTFSI solution in MeTHF. This electrolyte was chosen due to the partial solubility of the copolymers in organic electrolytes. The cyclability might actually be better, but decreasing the cut-off voltage may damage the integrity of the organic electrodes.

Poly(anthraquinonyl imide)s (PAQIs) that were synthesized from pyromellitic dianhydride or from 1,4,5,8-naphthalenetetracarboxylic dianhydride (NTCDA) and 1,4-diaminoanthraquinone (or 1,5-diaminoanthraquinone). This material was tested in a cell with NaPF_6_ that was dissolved (saturated, 0.5 mol L^−1^) in a mixture of dimethoxyethane (DME) and dioxolane (DOL) 1:1 by volume was used as the electrolyte. It delivered a capacity of 190 mAh g^−1^ and a stable cyclability with 93% capacity retention over 150 cycles in the range 1.5–3 V at current density 50 mA g^−1^ as a cathode in a sodium-battery [184]. This capacity, which is one of the largest that was obtained with an organic compound, corresponds to 3.4 electrons per molecular unit, which confirms the possibility that these polymers undergo a reversible four-electron redox reaction with simultaneous Na^+^ insertion/extraction [179]. However, to obtain this capacity, the electrode contained only 40 wt.% PAQI, 40 wt.% carbon, and 20 wt.% PVDF.

Xie et al. synthesized the dimers of NTCDA and 3,4,9,10-perylenetetracarboxylic dianhydride with benzodiimidazole as the connecting part between the monomers [185]. Much larger aromatic rings were formed after elongating conjugation, which led to improved thermal stability, but the price was a penalty on the gravimetric capacity.

NTCDA-derived polyimide (PNTCDA) was proposed as the anode material for an aqueous rechargeable lithium-ion battery (ARLB) [186]. With the LiCoO_2_ counter-electrode, this ARLB achieved a specific capacity of 71 mAh g^−1^ and a specific energy density of 80 Wh kg^−1^ in 5 mol L^−1^ LiNO_3_ solution at 100 mA g^−1^. PNTCDA was also tested as an anode for aqueous sodium batteries, but the performance was not as good, which was mainly because of the choice of the cathode. On the other hand, it delivered a discharge specific capacity of 140 mAh g^−1^ at an average potential of 2 V vs. Na^+^/Na, with a 90% capacity retention over 500 cycles at 1C (140 mA g^−1^) in a sodium-ion battery [187].

**Figure 6:**
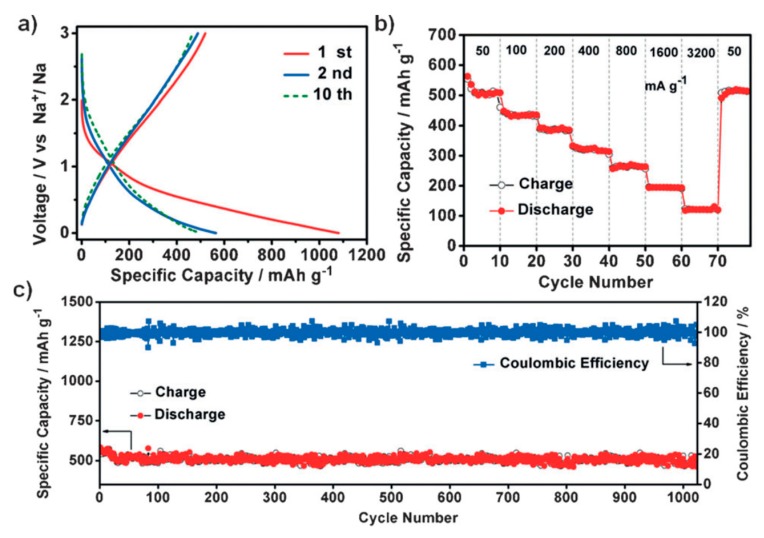
Electrochemical performance for polydopamine (PDA) synthesized by using (NH_4_)_2_S_2_O_8_ (APS) with APS/DA ratio 2:1, as sodium-ion battery anode: (**a**) Discharge/charge profiles at a current density of 50 mA g^−1^. (**b**) Rate performance. (**c**) Long-term cycling profiles at a current density of 50 mA g^−1^ (Reproduced with permission from [191]; copyright 2016 Wiley).

In an alkalyne environment, dopamine can be oxidized to 5,6-indolequinone before polymerization [188], with a molecular structure that is similar to benzoquinone and naphthoquinone, which have two redox-active quinone groups that store Li^+^- or Na^+^-ions [86,96,189]. In addition, the DFT computations predict a high redox potential of 2.83 V vs. Li^+^/Li for the polymerized form of 5,6-indolequinone [190]. These findings suggest that polydopamine is a promising carbonyl-based cathode for rechargeable batteries. A breakthrough in the use of polydopamine (PDA) was recently achieved by Sun et al. [191]. A higher o-benzoquinone content in PDA could provide more redox-active sites, since dopamine is oxidized to o-benzoquinone by electron transfer and the oxygen atom in o-benzoquinone is suitably located for coordination to the lithium/sodium ions [39,192]. However, this would be at the expense of the electrical conductivity. Therefore, a compromise must be found. This led Sun et al. to investigate the optimization of the crucial oxidation state for PDA in sustainable and biocompatible electrodes. A series of samples with APS/PDA ratios varying from 1:1 to 4:1, which were labeled as O-PDA-1 to O-PDA-4, respectively, where APS is ammonium persulfate (NH_4_)_2_S_2_O_8_ used for the synthesis of PDA was evaluated to determine the degree of oxidation. The optimized APS/DA molar ratio was 2:1, and the corresponding PDA-electrode delivered a capacity of 1818 mAh g^−1^. Given the adhesive ability of PDA, the cathode was composed only of PDA and conductive acetylene black with a weight ratio of 8:2, and no additional binder. At a current density of 500 mA g^−1^, the capacity was 1818 mAh g^−1^ for LIBs and 500 mAh g^−1^ for SIBs), and good stable cyclability (93% capacity retention after 580 cycles for LIBs; 100% capacity retention after 1024 cycles for SIBs). The electrolytes were 1 mol L^−1^ LiPF_6_ or 1 mol L^−1^ NaPF_6_ in EC:DMC (1:1 by *w*/*w*). These results show the remarkable stability of this material and the total absence of dissolution in the electrolyte (see Figure 6). Furthermore, partial oxidization plays a key role in balancing the proportion of the redox-active carbonyl groups and conductivity. In addition, the fact that the content of active PDA material was high, 80 wt.% of the electrode, is exceptional in the organic electrodes.

## 4. Salts

### 4.1. Quinone Based Salts

Another strategy envisioned mitigating organic-electrode dissolution in non-aqueous electrolytes is to use organic salts, since they are intrinsically less soluble than the small organic molecules [20,24,95,128,138,193,194]. However, for organic salts the coordination bond, such as O···Li···O, while preventing the dissolution of the active material from dissolving, the long-term cycling stability is never satisfactory. This led Song et al. to synthesize the lithium salt of poly(2,5-dihydroxy-p-benzoquinonyl sulfide) (Li_2_PDHBQS) [37]. This oligomeric lithium salt combines the merits of both organic salts and polymer electrode materials to utilize the fast kinetics of the BQ group and the stability of the amorphous structure. As a cathode (polymer, carbon, binder in the ratio 6:3:1) for a Li-ion battery, with LiTFSI solution in a mixed solvent of DOL:DME (1:1 by *v*/*v*), it delivered a capacity of 268 mAh g^−1^. At 500 mA g^−1^, the capacity retention was still above 90%, and even after 1500 cycles relative to the second cycle (239 mAh g^−1^). Therefore, dissolution in the electrolyte was completely removed; a problem is the low operating voltage. Cyclic voltammetry curves show the two peaks at 2.05/2.19 V and 1.89/2.03 V vs. Li^+^/Li.

### 4.2. Azo Based Salts

A new type of organic electrode material based on the azo group (–N=N–) was recently proposed for large-scale SIBs. The azo group functions as a redox center to reversibly react with Na-ions. In particular, azobenzene-4,4’-dicarboxylic acid sodium salt (ADASS) provides 170 mAh g^−1^ at 0.2C and it retains 66% and 58% capacity when the current density increases to 10C and 20C, respectively [195]. Moreover, a reversible capacity of 98 mAh g^−1^ is retained for 2000 cycles at 20C, with a slow capacity decay rate of 0.0067% per cycle. This performance is due to the addition of a carboxyl group that avoids the dissolution of azobenzene in the electrolyte (1 mol L^−1^ NaPF_6_ in diethylene glycol dimethyl ether (DEGDME)). The charge/discharge equilibrium potentials of ADASS are 1.37 V/1.25 V. In terms of power density and cycle life, this azo-based anode outperforms the other organic anodes for sodium-ion batteries, only based on carbonyl (C=O) compounds, such as sodium terephthalate [135,143] and benzoquinone derivatives [123,196], and imine (C=N) compounds, such as Schiff base derivatives [197]. However, the doping-reaction-based organic materials [198], such as organic radicals, display high reaction potentials during the p-doping process with the anions of the electrolyte [199], and are thus not suitable as anodes.

Azo-based organic electrodes are also very promising for LIBs [200]. During initial electrochemical lithiation, the nitro group in crystalline 4-nitrobenzoic acid lithium salt (NBALS) is irreversibly reduced to an amorphous azo compound. In 7 mol L^−1^ LiTFSI in 1,3-dioxolane: dimethoxyethane (DOL:DME) electrolyte, with the initial capacity 153 mAh g^−1^ at 0.5C. Subsequently, the azo compound was active and a capacity of 131 mAh g^−1^ after 100 cycles at 0.5C was delivered. In the same work, the authors replace NBALS with another azo-compound: 4-(phenylazo) benzoic acid lithium salt (PBALS), which remains crystalline upon cycling. The extended π-conjugation in PBALS and the strong Li-ion adsorption by nitrogen in the azo group contribute to fast Li-ion diffusion. At 10C, the reversible capacity is 95 mAh g^−1^ for 500 cycles with a very slow capacity decay rate of 0.032% per cycle, even though the size of the particles is large (1 μm). The two discharge plateaus are at 1.47 and 1.25 V. Again, this result reflects a favorable crystal structure to avoid the dissolution of the organic electrode and to obtain excellent cycle life. The azo compounds with extended π-conjugation are promising anode materials for LIBs, because the voltage is below 1.5 V.

## 5. Nitroxide Radical Polymers

The recent interest in nitroxide radical polymers has led to several remarkable breakthroughs in diverse fields, such as medical imaging and energy storage applications. The synthesis strategies that are available for their preparation were reviewed [201].

Cathodes that were based on poly(2,2,6,6-tetramethyl-1-piperidinyloxy-4-yl methacrylate) (PTMA) were proposed, because the stable polymer contains repeating units of a nitroxyl radical that is known to exhibit very rapid electron transfer [54,202,203]. The nitroxide radical, 2,2,6,6-tetramethyl-1-piperidinyloxy (TEMPO), is the repeating unit, which has low molecular weight (C_9_H_18_NO *M*w = 156.2 g mol^−1^) per radical moiety and unpaired electrons that are almost fully localized on the radical. Compounds containing the TEMPO radical are known to show redox behavior in aprotic solvents in the range of about 3.7–4.0 V vs. Li^+^/Li at low rates. The PTMA cathode has a capacity of 111 mAh g^−1^ [204,205], but it suffers from the loss of contact between PTMA and the carbon mixture during cycling [206,207], irreversible side reactions at high voltages [208], and self-discharge [209]. A solution for improving the conductivity and the cyclability is to encapsulate PTMA in carbon nanotubes [210]. In a Na-cell with 1 mol L^−1^ solution of NaClO_4_ in ethylene carbonate/diethyl carbonate (1:1 by *v*/*v*), PTMA-impregnated CNT electrodes for a Na-ion battery delivered a capacity during the first discharge of 222 mAh g^−1^ at 0.1C, and it exhibited two notable voltage plateaus at 3.36 and 2.1 V, corresponding to the two-stage reduction of the oxoammonium cations to nitroxide radicals at the first plateau and of nitroxide radicals to aminoxy anions at the second plateau. The rate retention was 86% when the current density was raised to 5C, owing to the high conductivity of CNTs. The capacity retention was 93% after 100 cycles at 0.5C, which was proof that the CNT shell was effective in preventing the dissolution of PTMA in the electrolyte. An improvement was obtained by replacing the aluminum current collector with graphite, which makes it possible to eliminate the addition of carbon to the active material in the preparation of the electrode, and by replacing the EC:DEC electrolyte by a polyimide-based gel polymer to avoid dissolution [211]. The discharge capacity of the corresponding (PTMA-GS) cell was 184.8 mAh g^−1^ at a 0.5C-rate, with 88% retention after 100 cycles. At 10C, the capacity was still 72.3 mAh g^−1^, which is comparable to that of the PTMA-impregnated CNT electrode, being based on grams of active material. The difference comes the fact that, with PTMA-GS, the cell is binder-free and no carbon is added in the electrode, which contains 100 wt.% active organic material, as compared to at best 60 wt.% in most organic electrodes. The consequence is improved energy density, which is reported at 470 Wh kg^−1^ vs. cell weight in PTMA-GS.

Other molecular structures with the nitroxide radical were designed that have similar features with a characteristic electrochemical activity at ∼3.5 V vs. Li^+^/Li [212,213]. More recently, another molecular structure, 2,3,6,7,10,11-hexamethoxytriphenylene (HMTP), was proposed that has the same reduction–oxidation potential, and that did not experience self-discharge over one month to achieve >95% retention of the initial discharge capacity after 50 cycles at 1C in a lithium cell with 1 mol L^−1^ PF_6_ in EC:DMC (1:1 by *v*/*v*) as electrolyte [214]. To explain this result, the authors showed that the one-electron reduction of HMTP during discharge leads to the formation of a radical anion HMTP^•-^, in which the electron is delocalized over the entire molecule. This is in contrast to the nitroxide-containing molecules, where the charge is always localized. The delocalization of the electron stabilizes HMTP*, thus avoiding self-discharge. The drawback is the low capacity (~66 mAh g^−1^), which is close to the theoretical value.

## 6. Ferrocene Based Materials

Ferrocene (Fe(C_5_H_5_)_2_, C_5_H_5_: cyclopentadienyl) is reversibly oxidized by one-electron exchange. Ferrocene-based redox active colloids, paired with viologen-based redox active colloids, cycle efficiently and reversibly in a nonaqueous redox flow battery with a size-selective separator [215]. The microscale dimensions of these redox active colloids dramatically decrease crossover. The modular nature of redox active colloid synthesis can be used for other redox active pendant moieties.

## 7. Poly Fluorenylethynylene

Poly(fluorenylethynylene), i.e., a fluorenyl radical that is attached to a poly(acetylene) backbone derivatives, attracted attention to obtain cathodes working “p” doping [216,217]. A high degree of polymerization inhibits the dissolution in electrolytes. Moreover, the overlapping π-orbitals of alternating double- and single-bonds, π-electrons in conjugated polymers are easily moved from one bond to the other, which is beneficial to the rate capability [218]. Two of these poly(fluorenylethynylene) derivatives were tested [219] with propylene carbonate, 0.5 mol L^−1^ lithium perchlorate as the electrolyte. They delivered a capacity of 73 mAh g^−1^ at 1C, which is close to the theoretical value (82.3 mAh g^−1^); the other one delivered a capacity of 37 mAh g^−1^ at 10C that was stable over 100 cycles. The average operating voltage (3.5 V) is what can be expected for the formation of a carbocation and anion insertion, but the capacity is small, even though it is close to theoretical, because only one-electron transfer occurs for the fluorenyl moiety.

## 8. Flavin Compounds

**Figure 7:**
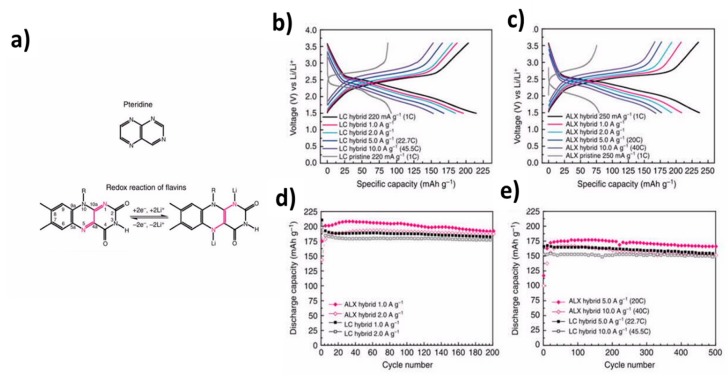
(**a**) Molecular structures of pteridine derivatives. The centre of redox reactivity in flavins is the isoalloxazine ring system, specifically the conjugated diazabutadiene (N5-C4a-C10a-Ni1) region. For riboflavine, R = CH_2_(CHOH)_3_CH_2_OH; (**b**) Capacity–voltage profiles of lumichrome (7,8-dimethylalloxazine, LC)-CNT hybrid electrodes (LC hybrid) at various current rates and a pristine LC electrode at a 1C rate. (**c**) Capacity–voltage profiles of alloxazine (ALX, without the methyl groups at C7 and C8 of LC)-CNT hybrid electrodes (ALX hybrid) at various current rates and a pristine ALX electrode at a 1C rate. Capacity retention of the LC hybrid and ALX hybrid (**d**) at 1.0 and 2.0 A g^−1^ for 200 cycles and (**e**) at 5.0 and 10.0 A g^−1^ for 500 cycles (Reproduced with permission from [223]; copyright 2014 Nature).

Lee and co-workers explored the possibility of using the energy storage mechanism of flavin redox cycling in mitochondria to lithium rechargeable batteries by testing a riboflavin electrode versus lithium [220]. Riboflavin (also known as vitamin B2) is a biochemical source for the redox-active moiety of flavin cofactors. The flavin redox reaction occurs during battery operation at the nitrogen atoms of diazabutadiene in the flavin molecules using two successive single-electron and Li^+^ transfer steps, as illustrated in Figure 7. The flavin-electrode in a cell with the standard electrolyte 1 mol L^−1^ LiPF_6_ in EC:DMC (1:1 in volume) demonstrated a capacity of 106 mAh g^−1^, which is equivalent to 1.49 Li atoms per unit formula between 1.5 and 3.8 V at a current rate of 10 mA g^−1^. Note that the electrode contained 50 wt.% of active material (30 wt.% carbon black and 20 wt.% polytetrafluoroethylene), while, in most cases, the organic electrodes typically contain 30 wt.% of active materials. The operating voltage for the first insertion of lithium in flavin occurs at 2.6 V, and the second one is at 2.4 V. To increase the gravimetric density, the ribityl group at the N10 site in riboflavin was replaced with a methyl group. The simpler molecule, called lumiflavine (LF) (Figure 7a), delivered a capacity 174 mAh g^−1^ with the same operating potentials. On the other hand, substitution with much stronger electron-withdrawing groups, such as cyano, is expected to increase the voltage [221]. However, the lumiflavine electrode exhibited a small capacity retention of ≈66% after 10 cycles. Liang et al. investigated the feasibility of scaling up the flavin-based organic materials for use in positive electrodes and determined the effects of an aqueous binder system on the structural stability of flavin-based organic materials [222]. However, the problem of poor cycleability was not solved, so further investigations should focus on polymerization or immobilization to improve the stability of the electrode. As we shall see in the section that is devoted to the carbon-organic molecule composites, immobilization by grafting on carbon can improve the cycle life. This is the result of the noncovalent π–π interaction between the graphene surface and the backbones of the polymers due to the conjugated aromatic rings.

Lumiflavine is a simpler molecule than riboflavine, but it is possible to simplify even more to obtain a minimal redox-active unit of the diazabutadiene redox couple, with the goal of reducing the weight of the molecule. The pteridine nuclei were obtained by sequentially removing the substituents, and the pteridine molecules were attached to the surface of carbon nanotubes (CNTs) [223]. The pteridine/CNT electrode in a Li-ion cell with 1 mol L^−1^ LiPF_6_ in tetraethylene glycol dimethyl ether (TEGDME) as the electrolyte delivered 533 Wh kg^−1^ in 1 h and 348 Wh kg^−1^ in 1 min., as well as high cyclability retaining 96% of the initial capacity after 500 cycles at 10A g^−1^ (Figure 7b). On another hand, the same electrode in a Na-ion cell with 1 M NaPF_6_ in diethylene glycol dimethyl ether (DEGDME), as the electrolyte exhibited a poor cycle life, which was due either to a non-optimized electrolyte, or to a too large volume expansion of the active materials upon the insertion of the rage Na^+^ ions. A flow battery was built, using a sodium salt of flavin mononucleotide, riboflavin-5’-phosphate sodium salt (FMN-Na), which serves as the active element in the negative electrolyte, and ferrocyanide/ferricyanide as positive electrolyte. Nicotinamide (NA), which is also known as vitamin B3, was added as a hydrotropic agent to increase the water solubility of the riboflavine [224]. The flow battery with 0.4 mol L^−1^ K_4_[Fe(CN)_6_] in 1 mol L^−1^ KOH (catholyte) and 0.24 mol L^−1^ FMN-Na and 1 mol L^−1^ NA in 1 mol L^−1^ KOH (anolyte) delivered a capacity of 5.03 Ah L^−1^ at a current density of 25 mA cm^−2^, which is close to the theoretical capacity of 5.36 Ah L^−1^, with an average discharge potential 0.96 V, which leads to an energy density 4.83 Wh L^−1^. The solubility of the hexacyanoferrates limits the performance. The capacity retention was 99% after 100 cycles. The peak power density was 0.16 W cm^−2^ at a current density of 0.3 A cm^−2^, which is greater than the all-vanadium RFB (0.12 W cm^2^ at 0.15 A cm^−2^).

## 9. Tetrathiafulvalene Derivatives

Tetrathiafulvalene (TTF) is a heterocyclic molecule C_6_H_4_S_4_ that is electron-donating molecule and is very often associated with TCNQ to form the TTF[TCNQ] salt, which has many applications in electronics. TTF exhibits two successive one-electron reversible redox processes at 3.1 and 3.5 V vs. Li^+^/Li, and it forms stable radical cations and dications with counter anion(s) from the electrolyte. As TTF dissolves in the electrolyte, TTF analogues are formed: 2,5-bis(1,3-dithiol-2-ylidene)-1,3,4,6-tetrathiapentalene (TTP) and 2,2′-bi(5-(1,3-dithiol-2-ylidene)-1,3,4,6-tetrathiapentanylidene) (TTPY). The best capacity, cycle life, and highest voltage were obtained with TTPY, which is due to the highly conjugated molecular structure, with connections between the three TTFs of TTPY [225]. However, the disadvantage is that these materials still dissolve in the electrolyte when in the highest oxidation state (+4 for TTP and +6 for TTPY). The synthesis and electrochemical properties of pentakis-fused TTF derivatives possessing two cyclohexene-1,4-diylidene-inserted TTF units were investigated to overcome this difficulty [226]. The tetrakis(methylthio) derivative was found to be less soluble in organic solvents, even in carbon disulfide. In a Li-ion cell with 1 mol L^−1^ LiPF_6_ in EC:DEC (1:1 or 1:5 by *v*/*v*) as the electrolyte, its first discharge capacity was 196 mAh g^−1^ at 0.2C, which corresponded to 92% of the theoretical value for a ten-electron redox process, with average voltage of 3.56 V. The higher voltage is due to the multi-fused TTF systems that are multi-electron donors. The initial energy density reached 700 Wh kg^−1^, which is much higher than that of TTPY (540 Wh kg^−1^). In addition, this material had a remarkable rate capability; at 100C, the capacity was still 64% of the capacity at 0.5C. However, capacity retention was still limited. The capacity was reduced by 20% after 30 cycles at 0.2C charge/0.5C discharge. Other derivatives of tris-fused TTF were proposed, but the cycling ability remained poor [227].

## 10. Porous Organic Skeletons

The geometrical arrangement of the organic material with a periodic porous two-dimensional (2D) structure deserves special attention, because the results are very promising. The naphthalene diimide undergoes a reversible two-electron redox reaction during lithiation and delithiation through an enolisation mechanism [44,48]. A molecule arrangement in a triangular form that is based on naphthalenediimides (NDI) constructed in cyclic structure was used to increase the capacity to 146.4 mAh g^−1^, which is close to the theoretical value at 0.1C and maintained at 71.1 mAh g^−1^ at a rate of 10C for 300 cycles [228] in a cell with 1 mol L^−1^ LiTFSI and 0.2 mol L^−1^ lithium nitrate in DOL:DME (1:1 *v*/*v*) as the electrolyte. The triangular geometry provides a large surface area that promotes high capacity. Moreover, the connection between each naphthalenediimide through cyclohexane rings improved the stability in the electrolyte and extended the capacity retention.

The same strategy with a porous organic skeleton was used [229]. In this case the polymeric skeleton was a honeycomb framework that consisted of benzene and triazine rings with a sheet-like morphology that contained both n-dopable and p-dopable regions, which are used either as an anode or cathode. The p-dopable and n-dopable regions have a potential range of 4.1–2.8 V and 2.8–1.3 V vs. Na^+^/Na, respectively. The pore size was 1.4 nm. This bipolar porous organic material (BPOE) has a high specific power of 10 kW kg^−1^ and specific energy of 500 Wh kg^−1^, where the weight is based on the mass of the organic electrode in a half Na cell, and the electrode in the half sodium cell contained 70% wt.% active material, 20 wt.% carbon black, and 10 wt.% binder (CMC). 80% of the initial capacity was retained after 7000 cycles, provided that the electrolyte was NaClO_4_ in PC (the capacity retention was very poor using NaClO_4_ in EC:DEC). These are the best results that are reported for a sodium-based energy storage cell. Other microporous organic polymers with similar structure as BPOE were synthesized. For example, a homo-coupled polymer with expanded-honeycomb structure, YPTPA, in which each monomer unit has 12 triphenylamines (TPA) units was used in an electrode of a Li-ion cell, with 1 mol L^−1^ LiPF_6_ in EC:DEC (1:1 by *v*/*v*) as the electrolyte. It delivered 97.6 mAh g^−1^ at 2000 mA g^−1^ with a capacity retention of 91.4%, owing to its high porosity and large surface area (1557 m^2^ g^−1^). The energy density was 334 Wh kg^−1^ and the power density 6816 W kg^−1^ [230]. This is the best result that was obtained from triphenylamine-based microporous organic polymers.

Covalent organic frameworks (COFs) are a class of crystalline porous polymers that integrate organic building blocks into ordered structures [231]. Yang et al. selected 1,3,5-triformylphloroglucinol (Tp) and 2,7-diaminobenzo[*lmn*][3,8]phenanthroline-1,3,6,8(*2H*,*7H*)-tetraone (DANT) as the reactants to generate chemically stable schiff base links naphthalimide-based porous COF [232]. Another COF was made by changing the linker from Tp to 1,3,5-triformylbenzene to adjust the conjugated backbone of the whole molecule. Schiff base formation from trifomyl phoroglucinol or triformyl benzene with naphtalene tetracarboxylic-diimide modified with —NH_2_ groups for the Schiff condensation made the COFs. The naphtalene imide moieties are active in the redox process, and the DANT molecule can also be written *N*,*N*’-bis 4,4’-anilino naphthalimide to make it clear. The difference with YPTPA is that their honeycomb-structured planes are stacked parallel to each other into a hexagon-rod shape.

Both of the COFs exhibited good rate performance and cycling stability as cathode materials for Li-ion batteries with 1 mol L^−1^ LiPF_6_ dissolved in a EC:EMC:DMC mixture (1:1:1 by *v*/*v*) as the electrolyte. Tp–DANT-COF delivered a reversible capacity of 71.7 mAh g^−1^ over 600 cycles at 7.5C, and Tb–DANT-COF showed an initial discharge capacity of 144.4 mAh g^−1^, which was close to the theoretical capacity. In addition, the performance without hybridization with supporting carbons, such as graphene or carbon nanotubes, was remarkable. This result confirms that the extent of the molecular conjugated backbone of the 2D COFs accounts for their electrochemical performance. The Tp-DANT-COFs demonstrated higher cycling stability when compared to the other porous polyimides, even at high charge currents [233].

Wang et al. produced 2D-layer nanosheets (3–5 nm in thickness) by the exfoliation of COFs [234]. The exfoliated COFs (ECOFs) benefit from shorter ion- and electron-migration length, thereby facilitating diffusion and contact with the redox sites of the organic molecules. They exfoliated anthraquinone-based COF [235] by condensation of the redox-active monomer 2,6-diaminoanthraquinone (DAAQ) with 1,3,5-triformylphluroglucinol (TFP) to form the 2D β-ketoenamine-linked DAAQ-TFP COF to prove the concept, t. The BET surface area of the exfoliated DAAQ-ECOF (5-nm thickness) was 216 m^2^ g^−1^ as compared to 450 m^2^ g^−1^ for the original COF. The DAAQ-ECOF was evaluated in a cathode that also contained super-P and PVDF (6:3:1) with Li metal counter electrode and 1 mol L^−1^ LiTFSI electrolyte in TEGDME. Two redox peaks were observed at 2.34 and 2.48 V, which corresponded to the reversible oxidization/reduction of the carbonyl groups. The reduction potential of DAAQ-ECOF was 0.4 V higher than that of the monomer (DAAQ), originating from the extended conjugation in the polymeric layer. After 70 cycles between 1.5 and 4 V at a rate of 20 mA g^−1^, the capacity was still 145 mAh g^−1^, comprising 96% of its theoretical capacity. The reversible capacity of the DAAQ-ECOF was 104 mAh g^−1^ after 1800 cycles, with a coulombic efficiency of 99% at a current density of 500 mA g^−1^. The capacity of DAAQ-ECOF was 107 and 76 mAh g^−1^ at 500 and 3000 mA g^−1^, respectively, which almost doubles and triples the values for DAAQ-TFP-COF at the same current rate. The limitation of electrochemical activity has two origins: in COF, the redox reaction kinetics are dominated by Li^+^-ion diffusion, while, in ECOF, it is a surface or near-surface effect that is dominated by charge transfer rather than ion transfer. Wang et al. [234] used 2,5-diamino 1,4-benzoquinone (DABQ), a smaller structural unit, to substitute 2,6-diamino-anthraquinone (DAAQ) on the edges of DAAQ-TFP-COF to increase the theoretical capacity. The capacity of the exfoliated structure DABQ-ECOF showed a discharge voltage that was 0.5 V higher than that of DAAQ-ECOF, and the capacity increased to 210 mA g^−1^. The ECOFs have generally a larger capacity and much better rate capability than their COF parent, which is owed to a much larger Li diffusion coefficient. This shows that the low ionic conductivity is the main limitation for 2D COF electrodes.

Xu et al. [236] produced electrodes by growing DTP- andI-COF on conductive CNT wires in-situ, where DTP- andI-COF is a crystalline mesoporous polymer that contains redox-active naphthalene diimide walls, while the triphenylene knots and boronate linkages are electrochemically inactive [237]. The capacity retention of this COF in a cell with LiPF_6_ as the electrolyte in a mixture of EC:DMC (1:1 by weight) was remarkable (constant during 750 cycles at 2.4C) and the rate capability was excellent, due to the very high pore size of 5.3 nm. However, the capacity was rather small (67 mAh g^−1^), even though it corresponds to an efficiency of 82% for utilizing the redox-active sites in the DTP- andI-COF@CNTs cathodes. The reason is that the triphenylene knots and boronate linkages of DTP- andI-COF are electrochemically inactive and they add weight. In addition, the electrode contained DTP- andI-COF@CNTs active material and conductive carbon black (Super P Li, Timcal), and polyvinylidene fluoride binder (PVDF, Aldrich.) in the ratio 7/2/2 by weight on an aluminum plate, so that the capacity of the whole cathode was even smaller.

Porous metal–organic frameworks (MOFs) were investigated in parallel with COFs for applications in energy storage devices, which is discussed in a short review on the balance between porosity and electrical conductivity in MOFs/COFs [238]. MOFs are outside the scope of the present review regarding organic electrodes.

## 11. Organic Molecule-Polymer Hybridization

Rather than using polymerization to stabilize organic molecules, another strategy is to hybridize the active organic molecules with a conducting polymer, which has the additional beneficial effect of increasing the electrical conductivity. High capacities were obtained with a sulfur heterocyclic quinone (dibenzo(b,i)thianthrene-5,7,12,14-tetraone = DTT) and a conductive polymer [poly(3,4-ethylene dioxythiophene):poly(styrenesulfonate) = PEDOT:PSS) that is used as a binder [239]. PEDOT with π-conjugated thiophene rings carries positive charges that are balanced by the dissociated sulfonate groups in PSS. DTT and PEDOT:PSS interact with each other because of the electron transfer of extended π-conjugated configuration of DTT (electron donor) and cationic PEDOT(electron acceptor), which results in a non-covalent interaction. This prevents the active DTT from dissolution. Moreover, the incorporation of the sulfur atom raises the initial reduction potential to 2.89 V, which is 0.3 V higher than that for its carbon counterpart 5,7,12,14-pentacenetetrone. DTT bends when lithium atoms insert and, in such geometry, every lithium atom coordinates with two oxygen atoms to strengthen the binding with DTT, so that DTT can efficiently take up four lithium ions. This material delivered a capacity of 292 mAh g^−1^ for the first cycle, and 266 mAh g^−1^ after 200 cycles at 0.1C, with the optimized choice of LiTFSI in DOL/ DME electrolyte and 1 wt.% LiNO_3_ in the electrolyte. However, even though the capacity was larger, the rate capability was significantly lower than in other quinone-based electrodes [114]; at 1C, the capacity was 220 mAh g^−1^. PEDOT:PSS is also associated with another polymer, poly(vinyl carbazole) (PVK), which is redox-active and based on the N-containing hetero ring to its radical cation at 4.0 V vs. Li^+^/Li [240]. This result confirms that PEDOT:PSS is an efficient binder that guarantees enhanced electrochemical performances of organic electrodes. PEDOT/thiophene-grafted graphene oxide (PEDOT/Th-GO) composites from covalent linking of Th-GO with PEDOT chains that are prepared by in-situ chemical polymerization displayed a higher degree of conjugation than PEDOT [241]. This electrode material (50 wt.% Th-GO) in a supercapacitor produced a capacitance of 320 F g^−1^ at a current density of 1 A g^−1^ with a capacitance retention of 80% at 1 A g^−1^ after 1000 cycles.

Increased charge storage capability was also obtained by hybridizing the quinone derivatives with a conducting polymer, such as polypyrrole [242] or covalent organic framework [235,243]. Poly(pyrrole-co-(sodium-3-(pyrrol-lyl) propanesulfonate)) (PP-PS) was obtained by chemically grafting the sulfonate anions on the polypyrrole chains. The redox reaction of the polymer occurs by the insertion and extraction of ionizable Na^+^ cations, making the copolymer a polymeric Na host cathode material [244]. This is a major difference with other redox-active polymers that were proposed for Na-ion batteries, in which the redox mechanism takes place through a p-doping–dedoping mechanism of large electrolyte anions [143,245,246]. In this case, the capacity is limited by low doping, and the rate capability is limited by poor kinetics, due to the large size of the migrating anions. The Na/PP-PS cell using 1.0 mol L^−1^ NaPF_6_ + EC:DEC (1:1 by *v*/*v*) electrolyte showed an average discharge potential of 3.3 V. The capacity that was delivered at 40 mA g^−1^ was 85 mAh g^−1^, quite steady over 100 cycles.

**Figure 8:**
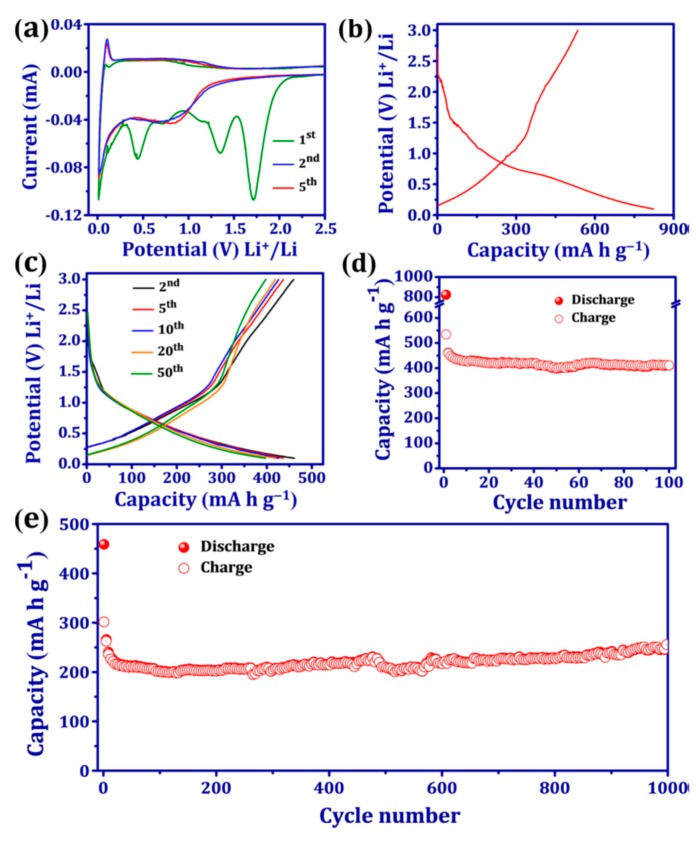
(**a**) Cyclic voltammograms of polyaniline (PANI)-squaric acid (SA) at a scan rate of 50 μV s^−1^ with respect to Li^+^/Li. (**b**) First cycle charge-discharge profile of PANI-SA within the potential range of 0.1–3.0 V vs. Li^+^/Li) at 100 mA g^−1^. (**c**) Charge-discharge profiles of PANI-SA for 2nd, 5th, 10th, 20th and 50th cycles at 100 mA g^−1^. (**d**) Cycling performance of PANI-SA at 100 mA g^−1^. (**e**) Long-term cycling performance of the PANI-SA electrode at 1 A g^−1^ current rate (Reproduced with permission from [247]; copyright 2018 Wiley).

In a different approach, 3,4-dihydroxycyclobut-3-ene-1,2-dione, which is also called squaric acid (SA), contains two C=O groups and two –OH in conjugation, was coated with another conductive polymer, polyaniline (PANI), through in-situ chemical polymerization. The PANI coat was very efficient in the dissolution of SA. The PANI-SA composite anode in a LIB with 1 mol L^−1^ LiPF_6_ in (1:1 by *v*/*v*) mixture of EC:DMC, as the electrolyte exhibited an initial reversible discharge capacity of 258 mAh g^−1^ at the second cycle and 250 mAh g^−1^ after 1000 cycles at 1 A g^−1^ [247] (see Figure 8).

## 12. Super Lithiation in Organic Electrodes

Except for nitroxides, the redox groups must be connected to a conjugated-saturated system to ensure the reasonable conductivity of the organic molecules, which lowers the energy density if they do not participate in the redox process. However, few organic materials can deliver capacities corresponding to a (C + N + O)/Li ratio close to 1/1 [125,248,249,250,251,252,253], and is the same when Na is used instead of Li. In particular, 3,4,9,10-perylene-tetracarboxylic acid-dianhydride was able to insert either 24 lithium ions or 15 sodium ions [123,125].

The common feature to all of these materials that are active by a super-lithiation (sodiation) process is the very low cycle and rate capability, which hinders their utilization, despite their very high initial capacity. However, dilithium benzenedipropiolate was identified as a new material of this class [254]. In addition to the reduction of the carbonyl groups, the unsaturated carbon-carbon bonds are reduced and reversibly oxidized to a Li/C ratio of 1/1. Consequently, an initial capacity of 1363 mAh g^−1^ was obtained when cycling at a very low rate (1Li/50 h) in the potential range 0–3.5 V with 1 mol L^−1^ LiPF_6_ dissolved in DEC:EC (1/1 by *v*/*v*) (LP40), but the capacity quickly decreases upon cycling. The cycle life is improved by reducing the voltage range to 0.5–3.5 V, in which case the capacity is stabilized at 180 mAh g^−1^ at a current corresponding to 1 Li/5 h.

## 13. Carbon Organic Molecule Composites

### 13.1. In-Situ Carbon Incorporation

Even if graphene is the best option at the laboratory scale, it is still very expansive and a more scalable composite is desirable. In this context, activated hierarchical porous carbon (a-HPC) is interesting due to its high surface area that is able to absorb a large quantity of active molecules. In addition, it has another advantage: the confinement effect of pores for a-HPC is an effective strategy for suppressing the dissolution of active materials and improving the cycling stability. Jaffe et al. covalently functionalized the sp^2^ surfaces of conductive carbon blacks by spontaneous reaction with diazonium salts of redox-active quinones [115]. The high-surface-area Ketjenblack (KB, BET surface area: 1220 m^2^ g^−1^) that was covalently functionalized with PAQ (PAQ = 9,10-phenanthrenequinone) delivered a capacity per total mass of quinone plus carbon of 75 mAh g^−1^ at a cycling rate of 30 mA g^−1^, and 58 mAh g^−1^ at a current density of 1500 mA g^−1^ with 1 mol L^−1^ LiTFSI electrolyte in propylene carbonate. The energy efficiency (charge/discharge) reached 96.1% with high energy density and power density over 80,000 W kg^−1^ for 500 cycles. These are the highest energy densities at high power that were reported with quinone electrodes. Even better results were obtained by substitution of PYT (PYT = pyrene-4,5,9,10-tetraone) for PAQ, which increased the electrode energy density at 75 mA g^−1^ from 160 to 300 Wh kg^−1^. This result is another piece of evidence that the porosity plays a positive role in facilitating access to the redox centers, and as they are tethered do not translate into more solubility. However, some solvent may also enter the porosity, and its weight must be taken into account when evaluating the full cell.

CMK-3 was used as a conductive matrix to encapsulate AQ [255]. Using 2 mol L^−1^ LiTFSI in DOL:DME electrolyte with 1% LiNO_3_ additive, the nanocomposite delivered an initial capacity of 205 mA g^−1^ at 0.2C and 84.9% capacity retention after 100 cycles, as well as good rate capability (146 mAh g^−1^ at 2C) in an ether-based electrolyte. This strategy was extended to other organic carbonyl compounds with biphenyl quinone substructures, such as BHNQ, 4,4′-dimethyl-1,1′-bi(cyclohexa-3,6-diene)-2,2′,5,5′-tetraone (DBT), 5,5′-bibenzofuran-4,4′,7,7′-tetraone (BFT), and 2,2′-binaphthyl-1,1′,4,4′-tetraone (BNT); all of these electrodes with 30 wt.% MK3 delivered more than 200 mAh g^−1^ after 100 cycles at 0.2C. These results suggest that encapsulating is effective in reducing the dissolution of the organic molecules in the electrolyte. When the AQ molecules were encapsulated in the core region of SWCNTs, an improvement in cyclability was also observed [256]. However, in this case, significant capacity decay was observed in the first 20 cycles, which was attributed to quinone molecules that are not encapsulated and/or encapsulated quinone molecules near the edge of single-walled carbon nanotubes (SWCNTs).

Zhang et al. introduced a-HPC as scaffold to fabricate the activated hierarchical porous carbon supporting poly(1,5-diamino-anthraquinone) (PDAA) (a-HPC@PDAA) composite and suppressing the dissolution of PDAA by the confinement effect of pores, and also by generating nanostructured PDAA particles [257]. The positive electrode containing 80 wt.% composites, 10 wt.% acetylene black, and 10 wt.% binder, was tested with a Li metal counter electrode. 1 mol L^−1^ LiTFSI in DME:DOL (1:1 by volume) was used as the electrolyte. The CV curves only showed one plateau at 2.1/2.2 V. The second plateau at 2.5/2.6 V, observed in pristine PDAA due to the quinone group was not observed in the a-HPC@PDAA-based electrode, because the enhanced π–π electron interaction between PDAA and a-HPC causes the gradual transition of the amine reacting position. At 100 mA g^−1^, HPC@PDAA lost 20% of its initial capacity after 100 cycles (from 250 to 200 mAh g^−1^). Further, only little fading was observed and the capacity retention was still 70.4% after 2000 cycles.

An original melt-polymerization process for a TEMPO (2,2,6,6-tetramethylpiperi-dine-1-oxyl)-containing monomer was proposed in [258]. Vlad et al. exploited the fact that the monomer melts at a temperature that is lower than that of the onset of polymerization reaction, which thus enables access to a molten monomer phase, in which carbon was uniformly dispersed. Subsequent polymerization froze the structure, yielding a homogenous carbon–polymer composite. Attention was focused on poly(2,2,6,6-tetra-methylpiperidinyloxy-4-yl methacrylate) (PTMA). The corresponding composite at 1C (100 mA g^−1^) displays a capacity of ~90 mAh g^−1^ in a cell using 1 mol L^−1^ LiPF_6_ in a mixture of EC:DEC (1:1 by *v*/*v*) electrolyte. The rate performance depends on the carbon content, but, with only 10 wt.% carbon, the capacity at 10 C was 52 and 46 mAh g^−1^ after 1200 cycles. This performance was obviously not as good as in the case of PTMA/CNT and PTMA/graphite composites that were evaluated in supercapacitors, but still acceptable in LIBs, because of the cycle life.

Kim et al. proposed a new strategy with a nano-fibrous polymer (NFP) film that was fabricated by electrospinning PTMA [259], in which a 14 wt.% solution of PTMA, PVdF-HFP, and carbon black in a mixed solvent was used. The final product consisted of an interconnected network of fibers with a diameter distribution from 20 nm to 1 μm and an average diameter of 50 nm. The fibers mainly consisted of a carbon core that was coated with PTMA. The fully interconnected micron-sized pores in the film were filled with electrolyte. The NFP film was used as an electrode in a LIB without additional processing. It exhibited a 3.6 V potential profile. The capacities that were delivered at 10C (3.3 mA cm^−2^) and 50 C (16.5 mA cm^−2^) were 111 and 109.2 mAh g^−1^, respectively, with capacity retentions of 98.5% and 90% after 150 cycles at 5C and 10C, respectively. The electrolyte was 1 mol L^−1^ LiPF_6_ in EC:DMC (1:1 by *v*/*v*). The performance was attributed to the nanostructure that promotes fast ion transport through short diffusion pathways. In addition, the NFP film prevented the dissolution of active material and it increased the mechanical strength of the flexible electrode.

The most conductive (but also most expensive) forms of carbon are often used, i.e. carbon nanotubes or graphene [260]. Bachman et al. [261] described a new class of nanostructured organic electrodes for rechargeable energy storage systems that were assembled by oxygen-functionalized few-walled carbon nanotubes (FWNTs) matrices that incorporate electro–polymerized pyrene derivatives. These electrodes are pseudocapacitive in nature, as the charge storage mechanism is based on redox reactions that are confined near the interface between the FWNTs and the polymer. Pyrene and its derivatives were identified as ideal candidates that generate stable redox couples at high potentials over 3.5 V vs. Li^+^/Li. These active elements of electrodes in Li-ion cells with 1 mol L^−1^ LiPF_6_ in EC:DMC (3:7 volume ratio) as the electrolyte exhibited high energy density (∼350 Wh kg^−1^) and high-power density (∼10 kW kg^−1^), with little capacity degradation over 10,000 cycles (85% capacity retention).

### 13.2. Carbon Nanotubes Organic Molecules

Mixing carbon nanotubes and organic molecules improved the electrochemical properties; an example is presented in Figure 7. In particular, Figure 7b,c illustrate a case where the pristine organic molecules (flavins) are almost inactive, but became active when hybridized with CNTs.

The covalent functionalization of MWNTs with an anthraquinone derivative was investigated [262]. The 2-aminoanthraquinone was transformed into the corresponding diazonium salt and then grafted to the surface of MWNTs via the chemical reduction of the diazonium function. Charge/discharge tests on purified and MWNTs grafted with anthraquinone and MWNTs simply mixed with the anthraquinone, i.e., without covalent grafting. The results highlighted the fundamental importance of covalent grafting of MWNTs. In a cell with 1 mol L^−1^ LiTFSI dissolved in 50:50 TEGDME:DOL as the electrolyte, the grafted composite delivered a capacity of 110 mAh g_electrode_^−1^ when compared to a maximum capacity of 260 mAh g^−1^ for anthraquinone alone (i.e. without carbonaceous additives). Prolonged cycling at 50 mA gelectrode^−1^ showed that 80% of the initial capacity is retained after 500 cycles, with very limited change after 100 cycles. On the other hand, the electrode that was prepared with a mixture of MWNTs and anthraquinone molecules only delivered 25 mAh g_electrode_^−1^.

The construction of three-dimensional conductive networks of carbon nanotubes in carboxyl-based organic sodium trimesic (Na_3_C_6_H_3_(CO_2_)_3_, Na_3_TM) salt as microparticles in an anode of a cell where the electrolyte was 1 mol L^−1^ sodium perchlorate mixed with EC:PC (1:1 by volume) delivered a capacity of 214.6 mAh g^−1^ at 0.1 A g^−1^ and still 149 mAh g^−1^ at 1 A g^−1^. The capacity at 0.5 A g^−1^ was 168.2 A g^−1^, 89% of which was retained after 500 cycles, while the pristine Na_3_TM is almost inactive [263].

An alternative to only using polymeric materials is to combine a flexible radical polymer with a rigid organic framework. Geckeler et al. illustrated this concept by wrapping poly(2,2,6,6-tetramethylpiperidine-1-oxy-4-yl methacrylate) (PTMA) around SWCNTs [264]. The PTMA/SWCNT (4 wt.%) layer was fabricated by drop-casting on an indium tin oxide (ITO) substrate and was used as the positive electrode of a so-called organic radical battery, with a Pt wire and Ag/AgCl as the counter and reference electrode, respectively, in acetonitrile containing 0.1 mol L^−1^ tetrabutylammonium perchlorate (TBAClO_4_). In this half-cell, the composite delivered a specific capacity of 100 mAh g^−1^ at the current density of 1 A g^−1^. The operational voltage was 0.8 V (3.8 V vs. Li^+^/Li), which is in agreement with the redox potential of the pendant 2,2,6,6-tetramethylpiperidin-1-oxyl (TEMPO) group of the PTMA. This is 91% of the theoretical capacity, which indicates that almost all of the pendant TEMPO groups actively contributed to the reversible charge storage at this current density, while maintaining the transparency of the composite. For comparison, pristine PTMA only delivered 23% of the theoretical capacity at the same current density.

We previously mentioned that PTCDA was successfully tested as an electrode for SIBs. When tested as an electrode for Li-batteries, a PTCDA polymer showed a discharge capacity that was close to the theoretical value (127 mAh g^−1^), but with poor cycleability and rate capability [42]. However, 73% of the initial capacity is retained after 300 cycles at a current density of 100 mAh g^−1^ in a cell where the electrolyte was 1 mol L^−1^ LiTFSI in EC:DEC:DMC (1:1:1 by weight) when it is mixed with carbon nanotubes (CNT) [19]. Recently, a simple vacuum filtration process prepared PTCDA [265]. The corresponding PTCDA/CNT composite that was used as a free-standing flexible cathode on a current collector without binder delivered a capacity of 150 mAh g^−1^ at 50 mA g^−1^ for over 500 cycles in LIBs, and 57.8 mAh g^−1^ at 25 mA g^−1^ for SIBs. In addition, good rate capabilities (48 mAh g^−1^ at 2000 mA g^−1^ for LIBs and 48 mAh g^−1^ at 1000 mA g^−1^ with an average operating potential close to 2.5 V for SIBs were observed. For LIBs, the electrolyte was 1 mol L^−1^ LiPF_6_ in EC:DMC (1:1 by *v*/*v*); for SIBs, the electrolyte was NaPF_6_ in EC:DMC (1:1 by *v*/*v*).

The poly(3,4,9,10-perylene-tetracarboxylicacid-diimide with ethylene diamine)/carbon nanotube (PI/CNT) nanocomposite showed much better cyclability. With only 5 wt.% single-walled CNT, 93% of the initial capacity was retained after 300 cycles at 100 mA g^−1^ [19] in a cell where the electrolyte was 1 mol L^−1^ LiTFSI in EC:DEC:DMC (1:1:1 by *w*/*w*). However, the initial capacity (115 mAh g^−1^) was slightly smaller than that of PTCDA/CNT (127 mAh g^−1^), because PTCDA has a smaller molar mass when compared to PI (refers to the repeating unit) [266]. Therefore, polymerization was efficient in stabilizing the structure of PI/CNT, while the CNTs were efficient in improving the conductivity.

Polymerizing the dianhydride component and quinone sub-structure together comprised the process of carrying out the condensation polymerization of pyromellitic dianhydride (PMDA) and 2,6-diaminoanthraquinone [267]. In this procedure, the carbonyl groups on the dianhydride component and quinone derivatives are electrochemically active. Moreover, this polymer combines the high capacity of the quinone unit and the high stability and redox activity of the polyimide unit. A binder-free flexible organic cathode film was further prepared with 30 wt.% single-walled carbon nanotubes (SWNTs) and it delivered a capacity of 190 mAh g^−1^ with good rate performance, as it maintained 120 mAh g^−1^ at 20C in a Li-ion battery with 1 mol L^−1^ LiTFSI in DOL:DME (1:1 by *w*/*w*). The capacity retention was 91.5% after 300 cycles at 0.5C. This cathode outperforms a previous composite of graphene network-supported polyimide that delivered 175 mAh g^−1^ with 82% retention after 150 cycles at 0.5C. The amount of active material was 80 wt.% in this latter case [268].

Wu et al. synthesized single-wall carbon nanotube (SWNT) films as a current collector, and in-situ polymerized polyimide (PI) as the active material in a geometry where PI nanoflakes were in intimate contact with one another and vertical to the SWNT film surface [171]. As a cathode in a Li-ion battery with 1 mol L^−1^ LiTFSI in DOL:DME (1:1 by *w*/*w*), this composite delivered a capacity of 226 mAh g^−1^ at 0.1C (1C = 443 mAh g^−1^) and it still delivered 120 mAh g^−1^. This remarkable rate capability is due to the nanosize PI flakes and the high conductivity of the SWNTs. The capacity retention was 85% after 200 cycles at 0.5C. Wu et al. also fabricated a large-area flexible polymer electrode by using a new type of polyimide-derivative pyromellitic diimide with ethylene diamine (PMTA)/SWCNT nano-cable composite [269], which was tested as a cathode that consisted of 65 wt.% active material, 30 wt.% conductive carbon, and 5 wt.% poly(tetrafluoroethylene) (PTFE) in a cell with the same electrolyte. A composite of PMTA/SWCNT with 10 wt.% SWCNT that was produced at 200 °C exhibited the highest capacity (163 mAh g^−1^) and optimal rate performance (124 mAh g^−1^) at 2C (1C = 383 mAh g^−1^). PMTA/SWCNT has an average discharge plateau of 2.09 V and average charge plateau of 2.21 V at 0.5C. The capacity retention was 86.6% after 200 cycles at 0.5C. The capacity of 160 mAh g^−1^ (based on weight of the composite) at 0.1C corresponds to 179 mAh g^−1^ that is based on pure polymer, whereas PMTA without SWCNT has a capacity of only 67 mAh g^−1^. Moreover, this PMTA/SWCNT flexible electrode showed 80% retention of the initial capacity after being bent 1000 times.

In a previous section we showed that polydopamine is a promising carbonyl-based cathode. Being used as a cathode, a porous network structure consisting of polydopamine coated FWNTs (few-walled carbon nanotubes) demonstrated a capacity of ~133 mAh g^−1^ in Li cells and ~109 mAh g^−1^ in Na cells when cycled at high voltage region from 2.5 to 4.1 V vs. Li^+^/Li. The polydopamine itself stored ~235 mAh g^−1^ in Li cells and ~213 mAh g^−1^ in Na cells [190]. In Li cells, the electrolyte was 1 mol L^−1^ LiPF_6_ in EC:DMC (3:7 by *v*/*v*). For Na cells, NaPF_6_ replaced the salt. This work illustrates the benefits of polymerizing redox-active molecules onto conductive carbon substrates [171].

Immobilization was the strategy that was chosen by Lee et al. to improve the cyclability of lumiflavine [92], since the aromatic structure of flavin molecules provides a strong anchor to the hydrophobic surfaces of the conductive single-walled carbon nanotubes (SWCNTs) scaffolds via π–π interactions. The non-covalent immobilization of the redox molecules via π–π interactions on the SWCNTs preserved the chemical structure of the redox-active diazabutadiene motif (N_5_-C_4a_-C_10a_-N_1_) in lumiflavine (LF), and the LF/SWCNT composite delivered a capacity of 204 mAh g^−1^ (1.95 Li^+^/molecule, 98% theoretical value), even at 1C rate (0.2 A g^−1^) in a cell with 1 mol L^−1^ LiPF_6_ in TEGDME as the electrolyte. Moreover, the cycling performance was drastically improved, since the composite showed a capacity retention of 99.7% over 100 cycles, which demonstrated that the dissolution of LF molecules in the electrolyte was effectively suppressed. Note, however, that the capacities are reported per gram of LF, while the composite only contained 45% LF.

### 13.3. Graphene Organic Molecules Composites

Graphene is the form of carbon with the highest electrical conductivity. The interest in the synthesis of composites of organic molecules with graphene is not only an increase of electrical conductivity. The strong physisorption of the molecules on carbon suggests that binding to a graphene sheet reduces the solubility of the organic compounds in the electrolyte solutions. This was predicted by Yu, who computed the adsorption of the 9,10-anthraquinone (AQ) and its derivatives, i.e., benzofuro(5,6-b)furan-4,8-dione (BFFD), benzo(1,2-b:4,5-b’)dithiophene-4,8-dione (BDTD) and pyrido(3,4-g)isoquinoline-5,10-dione on a graphene sheet by density functional theory with van der Waals dispersion-correction [270]. A similar study was also performed on phenanthraquinone (PQ), pyromellitic dianhydride (PMDA), and their derivatives, i.e., benzo(1,2-b:4,3-b’)difuran-4,5-dione (BDFD), benzo(1,2-b:4,3-b’)dithiophene-4,5-quinone (BDTQ), 3,8-phenanthroline-5,6-dione (PAD), pyromellitic dithioanhydride (PMDT), pyromellitic diimide (PMDI), and 1,4,5,8-anthracenetetrone (ATO) [271]. The binding energies of these molecules on graphene from weak to strong increased in the order BDFD < BDTQ < PMDA≤PMDI < PMDT < PQ < PAD < ATO. A first-principle analysis of the enhanced adsorption of carbonyl molecules on graphene via the π–Li–π interaction was conducted that is expected to also apply when Li substitutes for Na [272].

Yang et al. synthesized 2-aminoanthraquinone (AAQ) nanowires with an average diameter of 100 nm at 300 K through anti-solvent crystallization that was simultaneously attached to graphene oxide (GO) nanosheets. A flexible self-supported composite of AAQ nanowires that were wrapped in a 3D graphene network was obtained after chemical reduction [273]. The AAQ was chosen because the amino group is effective in controlling the crystallization, and it does not affect the theoretical capacity with respect to AQ. The composite was tested as a cathode in a lithium battery without any other conductive carbon or binder, where the electrolyte was 1 mol L^−1^ LiTFSI with 0.1 mol L^−1^ LiNO_3_ in DOL:DME (1:1 by *v*/*v*). It delivered a capacity of 265 mAh g^−1^ at 24 mA g^−1^ (0.1C), which is a remarkable result, since the capacity is per gram of electrode.

The π-conjugated quinoxaline-based N-containing heteroaromatic molecules (3Q), which were obtained by the condensation of cyclic carbonyl molecules with o-phenylenediamine, have a number of electron-deficient pyrazine sites, where multiple redox reactions take place [274]. A hybridized graphene electrode in an ether-based electrolyte had a discharge capacity of 395 mAh g^−1^ at 400 mA g^−1^ (1C) in the voltage range of 1.2–3.9 V, with nearly 70% capacity retention after 10,000 cycles at 8 A g^−1^. It also exhibits a capacity of 222 mAh g^−1^ at 20C, which corresponds to 60% of the initial specific capacity. Note the loading of active material in the electrode was 45 wt.%. These heteroaromatic molecules have multiple redox sites that are promising when coupled to graphene for high-energy-density batteries.

Vat dyes are commercially available carbonyl products that are readily obtained from plants or are artificially synthesized. A combination of sonication and hydrothermal process was proposed for scalable synthesis of vat dye/graphene oxide composites as organic cathodes for LIBs [275]. For example, Vat Green 8 (VG 8) contains a large condensed aromatic ring system with electroactive conjugated carbonyl groups. The vat dye was on the graphene layers with a weight ratio of dye to graphene of 0.5 (VG 8/G-0.5). Therefore, the strong anchoring of the vat dye on the graphene sheet from the π–π interactions promoted a stacking 2D structure with VG being intercalated between the graphene sheets. The vat dyes, which are not initially electrochemically active, became active in vat dye/graphene composites, and the strong interactions immobilized the carbonyl species on the conducting graphene sheet, inhibiting dissolution of the active molecules in the electrolytes, to extend cycle life. Finally, the high conductivity of graphene results in higher rate capability. The composite with VG 8/G-0.5 displays the best performance, with a capacity of ≈ 272 mAh g^−1^ without any decay in 200 consecutive cycles at a 100 mA g^−1^ and ≈ 74 mAh g^−1^ at 3200 mA g^−1^. The electrolyte was 1 mol L^−1^ LiPF_6_ in a mixture of EC:DEC:DMC (1:1:1 by *v*/*v*). This result illustrates the efficiency of the immobilization of carbonyl species on the conducting matrix/substrates, which is also a strategy also [25,115,276] with other forms of carbon.

A composite consisting of polyimide nanostructures that are vertically grown on dispersed graphene sheets (PI–FLEG) was synthesized in [277]. The synergetic effect of the high conductivity of graphene and the polymerization reduces the dissolution in the electrolyte consisting in 1 mol L^−1^ LiTFSI in DOL:DME (1:1 by *v*/*v*). The PI–FLEG composite electrode of a lithium battery delivered a capacity of 177 mAh g^−1^ at 0.1C. It retained 80% of its initial discharge capacity after 200 cycles at 0.5C, but the cycle life is still shorter than that obtained with the flavine-based electrodes. It is also important to note that the capacity was reported per gram of the active material, and its amount in the composite cathode was only 65 wt.%.

Graphene is an ideal matrix to support organic carbonyl compounds in flexible electrodes for SIBs, and a review on flexible SIBs was published recently [278]. Huang et al. prepared 3D graphene/polyimide composites by a one-step solvothermal process with simultaneous *in-situ* polymerization of PI on the graphene surface and self-assembly of graphene into a 3D network structure [279]. This material was evaluated as a highly flexible cathode without adding any binder or conductive carbon in a lithium-ion-battery (LIB), with 1 mol L^−1^ LiPF_6_ in EC:DMC (1:1 by *v*/*v*) as the electrolyte. The cathode delivered a capacity of 240 mAh g^−1^ at 40 mA g^−1^ and 102 mAh g^−1^ at 4000 mA g^−1^. The capacity retention was 82% after 600 cycles at 100 mA g^−1^. The reversible capacity of a SIB cathode was 213 mAh g^−1^ at 50 mA g^−1^ and an outstanding capacity retention of 80% after 1000 cycles at 1000 mA g^−1^.

A comparative study of graphene/polyimide and PAQS/graphite was reported [99]. Functionalized graphene sheets (FGSs) that were prepared by thermal expansion of graphite oxide were used to prepare the composites by simple in-situ polymerization on the graphene sheets. The capacity of polyimide (PI)-FGS-a (6 wt.% graphene) and PI-FGS-b (11wt.% graphene) increased to 172 and 205 mAh g^−1^, respectively, which corresponded to a polymer utilization ratio of 49 and 62%, respectively. The discharge capacity of PI-FGS-b at 10C was 68% of that at 0.1C, delivering 135 mAh g^−1^ in about 2 min., while pure PI had no electrochemical activity at this current rate. These results show that the electrochemical performance of the graphene/polyimide composite (60 wt.% active material-polymer + graphene) was much better than that of polyimide alone. Zhang et al. [280] observed comparable results with the PAQS/FGS composites in a cell with the same electrolyte, 0.1 mol L^−1^ NaPF_6_ in DME:DOL (1:1 by *v*/*v*). The self-assembled graphene/PAQS composite was prepared by the dispersion-assembly method and was used as a free-standing flexible cathode after mechanical pressing. The cathode delivered a capacity of 156 mAh g^−1^ at 0.1C (1C = 225 mAh g^−1^), which corresponds to 94.9% utilization of PAQS, and 102 mAh g^−1^ at 20C in a LIB. The reduction/oxidation potential was 2.07/2.52 V. This composite cathode in a SIB delivered a capacity of 157 mAh g^−1^ at 0.1C, which was based on the mass of the entire electrode, corresponding to a utilization ratio of 91% of PAQS, and 72 mAh g^−1^ at 5C. At 0.5C, the capacity retention was 71.4% after 1000 cycles. The two redox peaks are observed at 1.58/1.79 and 1.92/2.18 V at a scan rate of 1 mV s^−1^, so that the operating voltage is lower than in inorganic LIBs and the energy density will be lower in SIBs.

Disodium naphthalene dicarboxylate, Na_2_C_12_H_6_O_4_, nanoflowers wrapped in graphene were tested as an anode for a Na battery with NaPF_6_ dissolved in EC:DEC (1:1 by *v*/*v*) as the electrolyte, and yielded a capacity of 226 mAh g^−1^ at 0.1C (1C = 200 mA g^−1^) with a capacity retention of 92% over 100 cycles. The capacity at 10C was 88 mAh g^−1^ [281] and the voltage was 0.5 V vs. Na^+^/Na. Dilithium naphthalene dicarboxylate, Li_2_C_12_H_6_O_4_, was also tested as an anode for lithium batteries [16], and it showed a redox potential of 0.88 V. The cyclability of the electrode at high rate should also be good, owing to the π-extension of the core unit that separates the two redox active carboxylate groups. However, we are not aware of any results with Na_2_C_12_H_6_O_4_/graphene composite.

Compact two-dimensional coupled graphene and porous polyaryltriazine-derived frameworks with tailor-made pore structures (thickness 16 nm) were fabricated by using various molecular building blocks under ionothermal conditions [282]. The electroactive aromatic networks were immobilized on the graphene substrate by covalent bonding. They produced outstanding cyclability (395 mAh g^−1^ at 5 A g^−1^ for more than 5100 cycles), as well as excellent rate capability (135 mAh g^−1^ at 15 A g^−1^) that is due to the high electrical conductivity of graphene, in a LIB with 1 mol L^−1^ LiPF_6_ in 1:1 (*v*/*v*) mixture of EC:DMC. However, the capacity of 395 mAh g^−1^ was only gradually reached upon cycling; the capacity after 100 cycles was about 200 mAh g^−1^. The increasing capacity was attributed to the reversible formation and decomposition of an organic polymeric/gel-like film on the interface of the electrode materials that provides interfacial storage sites for excess Li^+^ through “pseudocapacitance-type behavior”. The voltammogram for the electrode is that of a supercapacitor and not that of an intercalation. Therefore, the data should be analyzed in terms of a supercapacitor, rather than that of a LIB.

Luo and co-workers tested croconic acid disodium salt for SIBs as nanowires [128], and they also wrapped CADS with graphene oxide by ultrasonic spray pyrolysis to enhance the conductivity of the CADS electrodes [283]. The GO-CADS composite was fabricated by ultrasonic spray pyrolysis to form sub-micron CADS particles that were encapsulated by the irregular shape (folds and wrinkles) of the graphene oxide. This composite delivered a high capacity of 293 mAh g^−1^, but the cyclability was not good, and was even worse than that of CADS alone. The reason is that, contrary to the usual case, the capacity decay with cycling is not due to dissolution in the electrolyte, but is due to the volume change from insertion/deinsertion of sodium that leads to the destruction of the particle’s integrity. The nanowires gave better results because of the nano-size that can easily accommodate volume change. However, this is an exception. When the cyclability is limited by dissolution, wrapping or coating the active particles by some form of conductive carbon usually limits the problem.

The sodium salt of poly (2,5-dihydroxy-p-benzoquinonyl sulfide)/RGO (Na_2_PDHBQS/RGO) composite was synthesized by incorporating 3.7 wt.% RGO at the initial of azeotropic distillation process of the synthesis of Na_2_PDHBQS [284]. The composite was made of Na_2_PDHBQS flakes that were uniformly grown on RGO, with 100–300 nm-thick holes in the flakes, which reduced agglomeration and increased the effective surface area. This polymer composite was tested as a cathode (60 wt.% Na_2_PDHBQS/RGO plus 30 wt.% carbon black and 10 wt.% PVDF) for SIBs. Two Na^+^-ions are reversibly inserted in Na_2_PDHBQS/RGO in one repeating unit, which corresponded to a theoretical capacity of 250 mAh g^−1^. The electrochemical tests were conducted in a narrow voltage range of 0.8–2.2 V in tetraethylene glycol dimethylether (TGM) plus 1 mol L^−1^ NaClO_4_, which gave better results than the carbonate ester-based electrolyte (1 mol L^−1^ NaClO_4_ in EC:DEC (1:1 by *v*/*v*), which provokes side reactions with the anionic group. The initial reversible capacity of Na_2_PDHBQS/RGO was 179 mAh g^−1^ at 100 mA g^−1^ (0.4C) and 183 mAh g^−1^ after 150 cycles, while that of the individual Na_2_PDHBQS polymers was reduced to 83 mAh g^−1^ and that of the sodium salt of the monomer, sodium chloranilate (Na_2_Cl), was only 14 mAh g^−1^ after the 150 cycles. At 4C, the capacity of Na_2_PDHBQS/RGO was still 150 mAh g^−1^.

A flexible binder-free composite with single-wall carbon nanotubes (PDHBQS–SWCNTs) was fabricated by vacuum filtration with SWCNTs to make a cathode for a Li-ion battery, with 1 mol L^−1^ LiTFSI in DOL:DME as the electrolyte [285]. This composite delivered a capacity of 182 mAh g^−1^ (0.9 mAh cm^−2^) at a current rate of 50 mA g^−1^ and a potential window of 1.5 V–3.5 V. The cathode still delivers 75 mAh g^−1^ (0.47 mAh cm^−2^) at 5000 mA g^−1^ and it retained 89% initial capacity at 250 mA g^−1^ after 500 charge–discharge cycles. The cell with a Li foil counter electrode retained almost 88% of its initial capacity after being bent 2000 times (bending radius 2.1 cm), which demonstrated a remarkable electrode material for use in flexible devices. The cathode did not contain any binder or conductive additive; the PDHBQS–SWCNTs was composed of 70 wt.% PDHBQS and 30 wt.% SWCNTs. The results show that PDHBQS is a promising molecule as an active cathode material for both the LIBs and SIBs.

Grafting electroactive materials onto insoluble materials is another strategy to eliminate the dissolution of organic molecules in the electrolyte. In particular, graphene nanoribbons (GNRs) were used as both a support and a charge carrier [286,287]. In-situ functionalization of 2,5-dimethoxybenzenediazonium salt and in-situ polymerization of 2,5-dimethoxystyrene yielded functionalized GNRs with protected 2,5-dimethoxyphenyl addends. The methyl groups that are protecting addend were then removed to yield redox-active GNRs [288]. However, the capacity was small even at slow rate (less than 35 mAh g^−1^).

Juglone, a renewable biomolecule that is derived from waste walnut epicarp, has a redox activity due to its quinone groups. In theory, a single juglone molecule can take up and reversibly release two Na atoms, leading to the theoretical capacity of 290 mAh g^−1^ as active material for SIBs with an operating voltage of 0.4 V. Juglone was immobilized on reduced graphene oxide (RGO) nanosheets by the strong π–π interaction between the aromatic structure and the carbon scaffold. In a SIB with 1 mol L^−1^ NaClO_4_ in EC:DMC (1:1 by *v*/*v*), the juglone/RGO composite electrode delivered a capacity that stabilized at 305 mAh g^−1^ after 10 cycles at a current rate of 0.1 A g^−1^, but it decreased to 280 and 212 mAh g^−1^ after 100 and 300 cycles, respectively. At 0.4 A g^−1^, the capacity was still 210 mAh g^−1^ [289]. An additional effect of RGO comes from the fact that RGO itself is an intrinsic anode with high capacity for SIBs [276]. Wang et al. also evaluated a juglone-RGO composite as an anode without additional binder or conductive agent and the Na_3_V_2_(PO_4_)_3_/C cathode in a flexible SIB [276].

## 14. Carbon Radical Organic Polymers

A methodology that combines the spectroscopic and thermal investigations of composites was proposed and applied to study the interactions of molecular components of the polypyrrole (PPy)–carbon system [290]. PPy, like polyaniline and most of the common conducting polymers, exchanges anions with the polymer backbone, which is sometimes described as a p-doping process. The anion insertion reactions can have high reduction potentials over 3 V, but the poor cycle life must be overcome. The combination of inter-penetrating CNTs with a good conducting polymer, like PPy, is useful because the strong interaction between carbon nanotubes and the NH-groups of PPy will result in improved mechanical strength and electrical conductivity from carbon contribution, especially when the polymer is in its reduced state. The bonding between the polymer and carbon depends on the chemical composition of the carbon surface. Bonding with PPy occurs by electronic π-stacking [291,292] from the attraction of p-orbitals of the electron-rich carbon aromatic rings with the electron-deficient orbitals of PPy for carbons that are highly graphitized and with minor surface functionalization [293]. On the other hand, with functionalized carbons, bonding occurs between the oxygen-containing functional groups on the carbon surface and the hydrogen from the –NH groups of the PPy rings [294]. However, the binding energy of π–π* stacking for the large molecules is much greater than the energy of hydrogen bonds, which explained the very stable structure with SWCNT-PPy, and the very weak structure with carbon black (CB)-PPy [290]. Poly(2,2,6,6-tetramethylpiperidin-1-oxyl-4-yl methacrylate) (PTMA), was also tested. Its electronic conductivity is nul, so an additive of conductive carbon is needed. The best conductive forms of carbon were used: graphene [181] and CNTs [264]. The results depended on the structural properties of CNT. Vertically aligned CNTs gave improved C-rate performance when compared to the randomly suspended CNT-PTMA composites, owing to an improved geometry of the CNTs that minimizes the electron path in the composite [295]. A capacity of 63 mAh g^−1^ was achieved at 100C with vertically aligned CNTs when compared with 49 mAh g^−1^ with suspended CNTs. In both cases, the electrolyte was 1 mol L^−1^ LiPF_6_ in EC:PC:DEC (3:2:5 by *v*/*v*) with 2% vinylene carbonate. However, the capacity at 1C was almost the same, namely 80–90 mAh g^−1^. It should also be noted that the contact or uniformity between the organic polymer and carbon in the composite electrode very much depends on the synthesis conditions and it is critical to insure good electrical conductivity of the composite [296].

PTMA was grafted on MWCNTs to form a MWCNT-g-PTMA composite with a compact core–shell morphology and a high active material loading of 60 wt.%. The capacity that was delivered at C/2 in a cell with 1 mol L^−1^ LiPF_6_ in EC:DEC:DMC (1:1:1 by *v*/*v*) as the electrolyte was 94 mAh g^−1^, which is 85% of the theoretical capacity, and decreased to 50% at 5C, much better than that of the control composite (non-grafted). At C/2, 87% of the initial capacity was retained after 200 cycles [297]. However, that the rate capability is not as good as that obtained on the vertically aligned CNT/PTMA composites, where the carbon was incorporated in-situ but not grafted [295]. It is worth noting that the conductivity of the grafted system (8.1 S cm^−1^) was actually less than that of the control composite (11.6 S cm^−1^) [297]. However, the electrochemical performance critically depends on the electrical contact between the carbon and active product, and the covalent bonding that is responsible for the grafting of PTMA on the nanotubes did not appear to facilitate electron transfer.

The best results from the different carbons, as expected, was obtained with graphene in PTMA@graphene, which delivered the highest reversible capacity of 222 mAh g^−1^ at 1C due to the high electrical conductivity. In addition, this composite also showed the best cycle performance over more than 20,000 cycles at 100C, due to the strong bond between PTMA and the graphene sheet [181]. The results demonstrate the improved capacity and rate capability promoted by graphene at with loadings as high as 60%.

## 15. Dual Ion Batteries

Dual-ion batteries operate by the incorporation of anions in the positive electrode and use the electrolyte as a source of these anions. Now, the most exemplified system uses graphite electrodes. However, the operating voltage for anion intercalation into graphite is above 4.5 V vs. Li^+^/Li and it exceeds the typical anodic stability limit of conventional electrolyte solvents (for a review on the salts and their solvents, see [10]). Ionic liquids can be used in this case, but they are still expensive. Consequently, efforts are underway to identify other organic electrode materials that function at lower potentials and are compatible with organic solvents in dual-ion batteries [298]. Coronene, which is a typical polycyclic aromatic hydrocarbon, was proposed [299]. The coronene electrode was tested in 1 mol L^−1^ LiPF_6_ in ethylene carbonate (EC) and diethyl carbonate (DEC) (1:1 by *v*/*v*) with a Li counter-electrode. The operating potential for incorporating PF_6_ was 4.0 V, but the capacity was limited to 40 mAh g^−1^.

However, 92% of the initial capacity was still retained after 960 cycles. Coronene has a crystalline structure, but the bonding between the molecules is due to weak van der Waals interactions, so the material becomes amorphous upon long cycling.

A few all-organic batteries using only redox polymers were proposed. The cathode utilized nitroxide radicals, either the TEMPO radical [266,300,301] or nitronyl nitroxides [302]. However, only the poly(2,2,6,6-tetramethylpiperidinyloy-4-yl acrylamide) and poly(viologen) systems exhibited reasonable capacities [300,301]. Thianthrene-based cathodes were also investigated [303]. The best result was reported with a poly(2-vinylthianthrene) derivative, which has a theoretical capacity of 110 mAh g^−1^ and a theoretical specific energy (440 mWh g^−1^) superior to that of PTMA (390 mWh g^−1^) [304]. For the anode of a full-organic cell, Wild et al. chose poly(2-vinyl 11,11,12,12-tetracyano-9,10-anthraquinonedimethane) (poly(2-vinyl-TCAQ)) that was proposed by Häupler et al. [305]. After polymerization, these materials were tested in electrodes that were prepared with Super P^®^ and PVDF, and an electrolyte consisting of 1 mol L^−1^ LiClO_4_ in EC:DMC (3:7 by *v*/*v*). At 1C, the reversible charge/discharge behavior was stable at a potential of 2.75 V for charging and 2.7 V for discharging. As the anode has a higher theoretical capacity (138.4 mAh g^−1^) than the cathode (110.6 mAh g^−1^), the capacity is based on the capacity of the cathode. After the formation of the SEI during the first cycle, the capacity stabilized and is constant at 71 mAh g^−1^ after 250 cycles.

A non-polymeric anion-inserting electrode, 5,12-diaminorubicene (DARb), was tested [306]. Rubicene contains an apolar moiety that intrinsically limits its affinity and tendency to be solvated by polar solvents such as common carbonate-based battery electrolytes. Hence, there is interest in coupling the rubicene core with the redox properties of amino groups. The electrode constituted of DARb with 33 wt.% carbon black was tested in a dual-ion cell configuration (vs. Li^+^/Li), with 1 mol L^−1^ LiPF_6_ in EC:DMC (1:1 vol.%) electrolyte. At a cycling rate of 1 electron being exchanged per diamino-rubicene unit in 5 h, an initial capacity of 115 mAh g^−1^ was obtained, and 75 mAh g^−1^ after 60 cycles with an average potential of 3.4 V vs. Li^+^/Li. This performance is still less than that obtained with the polymers mentioned above. 

## 16. Lithium-Sulfur Batteries

There is a tremendous activity worldwide to harness the sulfur electrode in Li/S batteries with expected major gains in specific energy. However, S_8_ is an inorganic material and it falls outside of the scope of this review (we have devoted a review to Li/S batteries elsewhere [307]). The main drawback of the sulfur positive is the formation of soluble polysulfides Li_2_S*_x_* (8 ≥ *x* ≥3) that act as a redox shuttle, resulting in low coulombic efficiency and lifetime. It has been suggested to react S_8_ with unsaturated double bonds, which is akin to vulcanization of rubber, to form C–S(S_y_)S–C (0 ≤ *y* ≤ 4) bonds in order to solve or minimize this problem. The preparation is quite simple and it can be scaled-up [308]. When reduced in an electrochemical cell, these materials lead to lower order polysulfides Li_2_S_y_ that are less soluble that the higher order Li_2_S_8_–Li_2_S_6_ or Li/S batteries.

Pyun et al. obtained, with the action of di-isopropenyl benzene on S_8_, polymers that kept a capacity > 1000 mAh g^−1^ after 100 cycles and that could be cycled further to 500 cycles. Alternatively, at higher temperatures, S can act as an electrophile on polymeric material, resulting in the formation of C–S bond and evolution of H_2_S [309].

A recent strategy consists in using oxygen atoms in organic molecules to fix the polysulfides by the formation of S–O bonds [310]. These authors synthesized a composite with AQ molecules that were adsorbed onto graphene. Both the experimental and the theoretical results showed that the sulfur in the polysuflides formed a S–O single bond and a S=O double bond with the oxygen in AQ. As a result, a LIB with this composite and LiTFSI in DOL/DME (1:1 by *v*/*v*) with 2 wt.% LiNO_3_ as the electrolyte delivered a capacity of 1013 mAh g^−1^ after 50 cycles at 0.05C. Improved results could be obtained by nitrogen-doping of the sulfur substrate with the same electrolyte, but without LiNO_3_ [311]. In this case, the Li-S cell with the electrolyte delivered a capacity of 800 mAh g^−1^ with a capacity retention of 95% after 100 cycles at a high current density of 0.7 mAh cm^−2^. In addition, this result was obtained with a high sulfur loading (4.2 mg S cm^−2^) and sulfur content (70 wt.%). This result led to the conclusion that the doping enhanced the adsorption ability of the polysulfides by the carboxyl group of AQ [312]. However, nitrogen doping is known to importantly modify the electrical properties of graphed, and it is also possible that this modification was at the origin of the improved electrochemical performance.

Excellent results over 1000 cycles with ≈ 80% capacity retention and 100% coulombic efficiency have been obtained with polymers, resulting from the action of S_8_ on poly(acrylonitrile) (PAN) [313]. If the results at this stage are very encouraging, then all of the materials in the sulfur chemistry suffer from a low operation voltage (1.8–2.5 V), which diminishes their interest.

## 17. Concluding Remarks

Organic electrode materials have rapidly caught the attention of the battery community in the past ten/fifteen years, as seen by the large number of publications on the subject, reflected in this review. The super capacitors have been only briefly touched, since an extensive recent review can be found in [314], and same for flow batteries [315]. Attention has been more focused of Li-ion, Na-ion, li-air or Li-O_2_, and Li-S chemistries. They have allowed scientists to show the ingenuity and creative imagination that is the hallmark of organic chemistry. The negative electrode candidates in the range 0–1 V vs. Li^+^/Li compete well with graphite, the workhorse in the field. At the positive electrode level, 3.5 volts seems to be near the upper limit for molecules/polymers that are working by cation insertion (“n” doping). To reach higher values (3.5–4 V), a “p” type doping is necessary, i.e., the insertion of anions that are compensated by carbocations (or a nitroxonium in the case of nitroxides radicals). This means that the electrolyte serves as reservoir for the salt, the cation of which inserts in the negative electrode and the anion in the positive one. The weight balance (Wh kg^−1^) that takes into account the weight of the electrolyte of the whole battery is lowered in this configuration, as compared with the insertion/desinsertion of the same cation in both electrodes, as in conventional LIBs.

While the capacities often surpass those of the inorganic materials, a less discussed aspect of organic materials is their lower specific gravity (1.5–2.2 g cm^−3^) when compared with ≈ 4.5 for LiCoO_2_ and NMC, for instance. This results in thicker electrodes, and/or a high fraction of the weight being allotted to current collectors.

Most organic electrodes work with considerable amount of conductive additive (carbon). It is unlikely that this can be reduced to the levels of today’s LiBs or SiBs (≈ 5%). Yet, this addition can be, could be diminished through optimization and the ad hoc choice of the carbon nature (nano-wires, graphene…) or conjugated polymer (PEDOT…). It has to be noted that several electrode materials that are presented here have extremely long cycle lives, which can account for the stability of the covalent bonds in organic materials

If organic-based electrode material may result on lower specific energy density, making them non-competitive for pure EVs, their advantages in terms of CO_2_ foot-print and fast kinetics suggest their use in grid storage and regulation, and possibly in HEVs.

## Figures and Tables

**Table 1:** Redox potentials calculated in H_2_O at pH = 7 for the most promising isoindole-4,7-dione derivatives defined by the substituents on the positions 2, 5, and 6 of Figure 2a (Me = methyl radical), after [88]. For the first isoindole-4,7-dione (IID) derivative, the potential 0.096 V is for the one-electron process. For the two other compounds, the potentials are reported for the two-electron processes according to the calculations.

Substituents			Calculated Redox Potential (V vs. SHE)
R^2^	R^5^	R^6^	
Me	CN	CN	0.096
Me	OH	OH	0.273
Me	CF3	H	−0.080

**Table 2:** Electrochemical characteristics, battery configuration and performance parameters of quinones versus other materials. * For the anode material. † Determined from three-electrode galvanostatic charge–discharge measurements. The electrodes of interest were placed at the positive side as the working electrode. Activated carbon cloth served as the counter electrode at the negative side. For pH −1 to 13, Ag/AgCl (0.197 V vs. SHE) served as the reference electrode. For more alkaline electrolytes, Hg/HgO (0.098 V vs. SHE) was used instead. ‡ For a battery consisting of anode/cathode materials and (if involved in the reaction) electrolyte. § The unusually long time for the small cycle number is due to the slow discharge (C/5) and charge (C/16) required for sustaining cycle life. MmH = nickel–metal hydride [9].

pH	Anode	Charge Carrier *	Reduction Potential *,† (V vs. SHE)	Specific Capacity * (mAh g^−1^)	Cathode	Specific Energy (Wh kg^−1^)	Energy Density ‡ (Wh L^−1^)	Cycling Stability ^a)^
−1	PTO	H^+^	0.51	395	PbO_2_	76	161	96%@1500 (1200 h)
−1	Pb		−0.34	129	PbO_2_	78	171	80%@240 (4500 h) §
−1	AC	H^+^	0.48	50	PbO_2_	38	37	83%@3000 (5500 h)
3~4	PPTO	Mg^2+^	0.04	144	CuHCF	25	45	66%@1,000 (1600 h)
7	PPTO	Li^+^	−0.06	229	LiMn_2_O_4_	92	208	80%@3000 (3500 h)
7	PPTO	Na^+^	−0.07	201	Na_3_V_2_(PO_4_)_3_	30	80	79%@80 (150 h)
7	LiTi_2_(PO_4_)_3_	Li^+^	−0.52	103	LiMn_2_O_4_	90	243	89%@1200 (1600 h)
7	Polyimide	Li^+^	−0.19	160	LiMn_2_O_4_	89	186	70%@50,000 (950 h)
13	PPTO	Li^+^	−0.06	195	LiCoO_2_	66	180	83%@700 (1200 h)
15	PAQS	K^+^	−0.60	200	Ni(OH)_2_	79	138	88%@1350 (2300 h)
15	MmH	H^+^	−0.81	300	Ni(OH)_2_	180	597	80%@1300 (n/a)
15	Zn	OH^−^	−1.19	500	Ni(OH)_2_	290	714	80%@300 (800 h)

^a)^ Capacity%@cycle number, (cycled time).
